# Cytoskeleton and Associated Proteins: Pleiotropic JNK Substrates and Regulators

**DOI:** 10.3390/ijms22168375

**Published:** 2021-08-04

**Authors:** Béatrice Benoit, Anita Baillet, Christian Poüs

**Affiliations:** 1 Université Paris-Saclay, INSERM UMR-S-1193, 5 Rue Jean-Baptiste Clément, 92296 Châtenay-Malabry, France; anita.baillet@universite-paris-saclay.fr (A.B.); christian.pous@universite-paris-saclay.fr (C.P.); 2 Biochimie-Hormonologie, AP-HP Université Paris-Saclay, Site Antoine Béclère, 157 Rue de la Porte de Trivaux, 92141 Clamart, France

**Keywords:** c-Jun N-terminal kinase (JNK), microtubule (MT), microtubule-associated protein (MAP), actin, actin-binding protein (ABP), intermediate filament (IF), intermediate filament-associated protein (IFAP), septin, septin-associated protein (SeptAP), spectrin, fodrin, endosomal sorting complex required for transport III (ESCRT-III)

## Abstract

This review extensively reports data from the literature concerning the complex relationships between the stress-induced c-Jun N-terminal kinases (JNKs) and the four main cytoskeleton elements, which are actin filaments, microtubules, intermediate filaments, and septins. To a lesser extent, we also focused on the two membrane-associated cytoskeletons spectrin and ESCRT-III. We gather the mechanisms controlling cytoskeleton-associated JNK activation and the known cytoskeleton-related substrates directly phosphorylated by JNK. We also point out specific locations of the JNK upstream regulators at cytoskeletal components. We finally compile available techniques and tools that could allow a better characterization of the interplay between the different types of cytoskeleton filaments upon JNK-mediated stress and during development. This overview may bring new important information for applied medical research.

## 1. Introduction

The cell cytoskeleton is a complex and dynamic set of networks essential for controlling cell shape, stiffness, adhesion, division, migration but also for stress sensing and cell adaptation to its environment. It is composed of four main players known as actin microfilaments, microtubule (MTs), intermediate filaments (IFs), and septin filaments (for review [1,2,3]). The building blocks of these filaments were discovered in 1942 (actin), 1967 (tubulin), mid-late 1970s (IFs), and 1971 (septin), and since then, new data on associated proteins, polymer regulation or functions are continuously generated (number of citations in PubMed: around 129,000 for actin, 82,000 for MTs, 19,000 for IFs, 1600 for septins). These four types of cytoskeletal filament-forming proteins assemble into distinct networks that are differently organized depending on cell types and cell cycle stages. Interestingly, novel crosstalks, which are mediated by a growing number of cytoplasmic proteins, keep being uncovered [1,4,5]. The cytoskeleton filaments are also indirectly linked to the extracellular matrix, to neighboring cells and to the inside of the nucleus, thanks to the ability of their components to bind cellular junctions and the nuclear envelope [6,7]. Note that at membranes also reside special cytoskeleton meshworks made of hexagonal spectrin arrays associated with short actin protofilaments or made of spectrin bundles associated with actin rings in neurons (for review [8,9]). Also, ESCRT-III proteins recruited by septins form filamentous spirals essential for inversed membrane fission and are thus now considered as a new element of the cytoskeleton ([10], for review [11,12]).

Along the years, the cytoskeleton has appeared both as a target and an organizing platform for a large array of cell signaling pathways, mediating the transport of several regulatory components or connecting the nucleus to the extracellular environment for example. Therefore, it appears as an effective stress sensor/integrator that can mediate adapted cellular responses (for review [13]). In this regard, the c-Jun N-terminal kinases and their upstream activators (MAPKKs and MAPKKKs) are key players in mediating cell responses to many abiotic and biotic stress insults, as well as in physiological developmental programs (embryos, neurons, immune cells) [14]. The three JNK genes (named *JNK1, 2, 3*, or *MAPK8, 9, 10*) encode 16 isoforms and mostly phosphorylate SP and TP consensus sites (SP/TP). The local activation of the kinase regulatory cascade, which in turn achieves the dual phosphorylation of a TPY motif carried by JNKs at position 183, generally depends on specific scaffolding proteins, which can be related to the cytoskeleton [14,15,16,17,18]. The extent and timing of JNK activation are also negatively controlled by MAPK phosphatases (MKPs), including dual-specificity phosphatases (DS-MKPs/DUSPs that dephosphorylate the TPY motif) [19].

In this review, we describe the organization of each cytoskeleton element and gather the mechanisms controlling cytoskeleton-associated JNK activation. We entirely cover the known cytoskeleton-related substrates directly phosphorylated by JNK, summarizing and supplementing the work of seminal reviews [15,20]. In a dedicated section, we point out specific locations of the JNK upstream regulators (including scaffolding proteins and dual-specificity phosphatases) at cytoskeletal components. Since other cytoskeletal substrates of JNK probably remain to be discovered due to their restricted locations and transient phosphorylations, we also present a series of available technical approaches that will help to uncover these substrates and decipher their effect on the cytoskeleton function and dynamics. Considering the increasing number of cytoskeleton-related proteins that continue to be evidenced, especially crosslinkers and coregulators, a more global view of the JNK effects on the cytoskeleton during stress and development should emerge in the near future.

## 2. JNK and Polarized Cytoskeletons: Microfilaments and Microtubules

**Figure 1:**
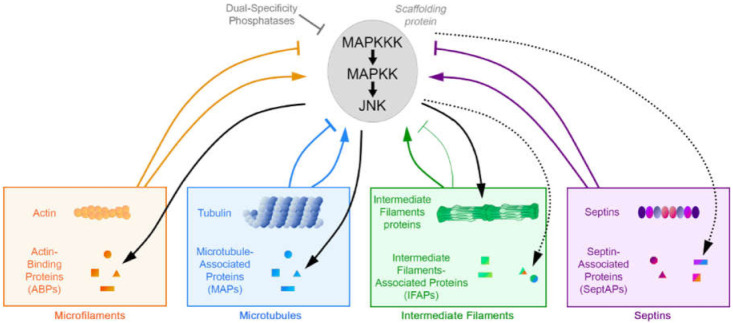
Relationship between the four main cytoskeletons and the JNK signaling pathway. JNK preferentially phosphorylates microtubule-associated and actin-binding proteins (MAPs, ABPs), as well as intermediate filament (IF) building blocks (black arrows). For IF-associated proteins (IFAPs) and septin-associated proteins (SeptAPs) only the ones also associating with MTs or F-actin filaments are known as putative substrates of JNK (dashed arrows). All the cytoskeletons can regulate the JNK signaling pathway (colored arrows).

Both microfilaments (F-actin) and MTs are polarized polymers, which exhibit dynamicity at their extremities (especially their “plus” end, also termed “barbed” end for microfilaments) and are able to auto-assemble. In cells, this self-assembly is regulated by a large set of dedicated proteins, the actin-binding and MT-associated proteins respectively (ABPs and MAPs, Table S1) [1,4], which regulate cytoskeleton dynamics, network formation, and remodeling, as well as control the interplay with diverse cellular structures. Among MAPs and ABPs, the molecular motor proteins drive the intracellular transport of several cargoes across the cytoplasm, some of them being cytoskeleton elements. For MTs, the transport can occur over long distances towards both “plus” or “minus” ends and is generally polarized, like in neurons, in migrating or dividing cells [21]. For actin, even if most myosin motors travel towards the “barbed/plus” end, the transport of cargoes is polarized in special structures like microvilli, stereocilia, and filopodia or at the leading edge of migrating cells (for review [22]). Figure 1 summarizes the main interlinks between the JNK signaling pathway, MT, F-actin, IF and septin cytoskeletons. The detailed overview of the literature is reported in Table S1 and completed by an extensive list of cytoskeleton-related proteins, with the indicated number of putative SP/TP phosphorylation sites that we found using the sequences of UniProt databank (column 5 of Table S1). So far, it appears that both F-actin and MTs can locally mediate JNK activation, but that cytoskeleton perturbations can also activate the stress kinase pathway. Furthermore, some MAPs and ABPs were identified as direct substrates of JNK (column 7 of Table S1), while tubulin and actin isoforms are not.

### 2.1. JNK and Actin Microfilaments

F-actin is composed of two protofilaments made of head-to-tail stack of globular monomers (G-actin). The resulting double-stranded helix of 7–10 nm diameter presents a “minus” (pointed) and a “plus” (barbed) end. The latter is more prone to incorporate ATP-bound actin monomers for polymerization, while reversely, the “minus” end depolymerizes by releasing actin in the ADP state. In cells, F-actin forms higher-order structures such as contractile (in myofibrils, cytokinesis rings) and non-contractile bundles (in stress fibers, filopodia, microvilli, invadopodia), 3D/branched meshwork (in pseudopodia, lamellipodia, endocytic invaginations) or periodic rings (in axons) [23]. ABPs and actin microfilaments are under the tight control of small GTPase proteins, including Cdc42, Rac and RhoA [24], which can, for example, orchestrate the reorganization of the cytoskeleton during migration or division (for review [25]).

#### 2.1.1. Control of JNK by F-Actin and ABPs

JNK can colocalize with F-actin and be activated at microfilament location, thanks to different ABPs. Indeed, Yang and colleagues [26] were able to visualize JNK proteins on actin stress fibers in human mesangial cells (HMC) and to coprecipitate JNK with the F-actin crosslinker α-actinin. They proposed that the actin cytoskeleton could function as a scaffold that tethers MAPKs and their activators. Indeed, MEKK1 (a MAPKKK of JNK) colocalizes with α-actinin along actin stress fibers [27]. The activation of JNKs can also be mediated by filamins, which are known to stabilize actin orthogonal networks and are considered to be JNK-pathway scaffolds (Section 5 for details) ([28,29], for review [16,30]). Consistently, the stabilization of actin microfilaments either by the RNAi knockdown of disassembly factors such as cofilin-1 or destrin, by the overexpression of the assembly factor WIP (WASP-Interacting Protein) or just by a phalloidin treatment, stimulates the JNK phosphoactivation [31,32]. Accordingly, transitory JNK binding to F-actin and activation occur upon fluid shear stress in endothelial cells and induce cytoskeleton remodeling [33]. Upon mechanical tension, Cdc42-controlled actin remodeling in alveolar stem cells also triggers JNK activation and pulmonary regeneration [34]. During development, accumulation to the actin cytoskeleton of the neuronal-specific JNK3 isoform upon palmitoylation results in reduced axonal branching, indicating the essential role of this JNK modification in axonal growth [35].

At the opposite, disruption of the actin cytoskeleton can also result in the activation of JNK through the MAPKKK DLK, which mediates axon regeneration ([36], for review [37]), or through stimulation of the MAPK pathway by the MST_1/2_/Ste20-like kinase commonly found on actin [38]. Interestingly some ABPs can also stimulate JNK activation when detached from F-actin. Among the S100 calcium-binding protein family, S100A1, A2, A4, A6, A8-A12, B, and P proteins are ABPs regulating the cytoskeleton organization and cell migration (for review [39]). However, when they are extracellular, some of these proteins also function as alarmins and in that case, their binding to the pattern-recognition RAGE receptors can mediate ROS/ASK1/JNK signaling activation and neuroinflammation (for review [40]). Also, when phosphorylated on Y353, ezrin of the actin/membrane-tethering ERM family, is released from the plasma membrane and F-actin in order to relocate to early and late endosomal compartments, where it recruits MKK7 and activates JNK [41]. Interestingly, the three ERM proteins (ezrin, radixin, moesin) activate Rac-1 and MEKK1/MKK4-MKK7/JNK in the presence of the adenoviral oncoprotein E1A [42].

#### 2.1.2. Phosphorylation of Actin Proteins and ABPs by JNK

So far, actin isoforms have not been found as direct substrates of JNK. Only the F-actin variant ACT-5/ACTB can be phosphorylated at Ser232 upon JNK3/KGB-1 activation in aged intestinal cells of *C*. *elegans* [43]. However, this serine, which does not belong to a SP/TP site (Table S1), may be phosphorylated by JNK only at a low rate or indirectly by a kinase under the control of JNK. Functionally, this phosphorylation disrupts the binding of the actin apical network to adherens junctions, weakening intestinal barrier activity. Since this serine is located in the troponin-binding site of ACT-5, its phosphorylation also impairs the binding of ACT-5 to non-muscle myosin [43].

A small set of ABPs has been shown to be JNK substrates, but to our knowledge, has not been extensively reviewed. The crosslinker MARCKS-like protein 1 (MARCKSL1) facilitates F-actin bundling, filopodia formation and prevents cell migration when phosphoactivated by JNK on Ser120, Thr148, and Thr183 [44]. The smoothelin-like-2 protein (SMTL2) is phosphorylated by JNK at Ser217, Thr236, Thr239, and Ser241, four sites located close to a docking D-site (JNK-binding domain/JBD, column 8 in Table S1) [15,45]. The phosphorylation of SMTNL2 could indirectly regulate Arp2/3 actin nucleation, since SMTNL2 can sequester coronin-1B and cortactin to allow Arp2/3 F-actin nucleation and cortical actin network stabilization [46]. The actin-capping protein CapZ dissociates from its partner CapZIP, when the latter is phosphorylated by JNK at Ser108 [47]. Caldesmon, which is known to prevent actomyosin contraction by binding to F-actin, is inhibited by its JNK-dependent phosphorylation at Ser789 [48]. More indirectly, JNK phosphorylation of the nuclear RhoGEF Net1A at Ser52 triggers its cytoplasmic relocalization and subsequent RhoA/ROCK/MYPT-mediated phosphorylation of myosin light chain (MLC2/MYL2) ([49], reviewed in [50]). In identical fashion to the LIMK kinase, JNK also contributes to F-actin realignment and enhancement of the endothelial barrier upon shear stress by phosphorylating the F-actin depolymerase cofilin-1/CFL1 at Ser3, resulting in its inactivation [51]. Surprisingly, a similar JNK-mediated phospho-inhibition of cofilin-1 has been involved in the abrogation of the epithelial barrier in endometrial cells, due to F-actin relocalization at tricellular contacts after cell treatment with the bacterial-derived toxin angubindin-1 [52]. Conversely, JNK inhibition by the classical SP600125 inhibitor rapidly increases transepithelial electrical resistance (TER, indicative of barrier integrity) of pancreatic cancer cells [53]. The *Drosophila* actin-nucleator p150-Spir is a JNK substrate, which harbors a JBD to dock the kinase (Table S1) [54]. The postsynaptic PSD95/DLG4/SAP90 protein, the function of which relies on α-actinin binding, is also phosphorylated by JNK at Ser295 [55]. Indeed PSD95/DLG4/SAP90, but also the postsynaptic shank3 protein are binding JNK3 [56]. VASP protein phosphorylations at Ser157 and Ser239, usually mediated by PKA/PKG kinases, are indirectly stimulated by JNK upon stress, leading to actin filament loss [57]. Moesin was also identified as an indirect substrate of JNK (not at a SP/TP site) in the process of podosome rosette formation [58].

Actin filaments are able to connect with focal adhesions (FAs) and adherens junctions (AJs) at the plasma membrane, which are both regulated by JNK (for review [59]). JNK directly phosphorylates paxillin on Ser178 to limit FA formation and allow fast cell migration [60]. This might depend on the MAPKKK MLK3 activity [61]. Additionally, the MAPKKK MEKK2 colocalizes with paxillin in FAs to induce its ubiquitylation and detachment from FAs [62]. The transglutaminase-2 (TGase-2/TG2), which stimulates paxillin phosphorylation at Ser178 is also an activator of the MAPKKK DLK (for review [63,64]), while the focal adhesion kinase (FAK) is able to phosphoactivate JNK at FAs through a p130Cas/Rac1/MKK4 pathway [65]. Concerning the adherens junctions, JNK indirectly favors the phosphorylation of catenin beta-1 at Ser33, Ser37 and Thr41 (not SP/TP sites) [66], which disrupts its binding to catenin alpha-1 and prevents adherens junction formation [67].

At last, a systematic search of docking sites for JNK suggested that more substrates remained to be characterized, such as the F-actin polymerases formin-1 and FHOD3, as well as the unconventional myosin MYO9B [15,68,69]. Also, docking sites for JNK are present in the shank1 and kank2 proteins [15,68,69], with respectively the Shank protein being a synaptic scaffold protein capable of regulating actin dynamic via cortactin and Arp2/3, and the kank protein being a focal adhesion component capable to bind both F-actin and MTs. Note that these five actin regulators contain several SP/TP sites, some of which being KSP/KTP sequences, known to be addedphosphorylated by JNK in CapZIP, but also in some polymers of neurofilaments (NFM, NFH) or in MAPs (CLIP-170, STMN3) (Table S1). Consistently, systematic phosphoproteomic analysis of purified neuronal growth cone samples revealed the presence of SP/TP phosphorylated sites, that could rely on JNK activity in several ABPs [70], such as formin-2, MYO5a, and shank1 (Section 6 and column 9 in Table S1).

### 2.2. JNK and Microtubules

A MT (for review [71]) is generally built of 13 protofilaments, each made of a head-to-tail stack of α/β-tubulin dimers. The resulting hollow tube of 25 nm diameter is characterized by dynamic instability of its extremities. In cells, the plus-end extremity, is generally free to incorporates GTP-tubulin dimers during polymerization phases and releases GDP-tubulin during depolymarization. During interphase, MTs are nucleated from the centrosome, but can also grow from the Golgi apparatus, from the nuclear envelope or from other MTs, while at the early phase of the mitotic spindle assembly, they are mostly nucleated in the vicinity of chromosomes and amplified through MT-based nucleation [72]. Dynamic MTs can scan the cytoplasm, allowing a process of search and capture of cell components. Dynamicity is also essential to build the spindle during cell division, while MT stability, which is characterized by a large panel of tubulin post-translational modifications (for review [73]), is important for transport in cilia or axons. Conversely, MT flexibility is required for flagella beating. 

#### 2.2.1. Control of JNK by MTs and MAPs

As observed with F-actin, JNK signaling can be activated at MTs. Indeed, active JNK colocalizes with neuronal MTs upon brain-derived neurotrophic factor (BDNF) stimulation [74]. Also, JIPs which are scaffolding proteins for the JNK signaling pathway (Section 5), are cargoes of the conventional kinesin motor on MTs, which supports a model where motor proteins spatially regulate the signal transduction pathways [75]. This interaction is responsive to cell stress to promote JNK activation since hyperacetylated MTs, generated either by cell starvation or by NaCl-mediated oxidative stress, recruit and stimulate JNK via increased binding of conventional motor kinesin to MTs [76,77]. In the case of starvation, this results in local Bcl-2 phosphorylation at Thr69, Ser70 and Ser87, leading to its dissociation from Beclin-1 and thus enhancing autophagy. In the case of oxidative stress, this results in local phosphorylation of the mitochondrial fission protein Drp1 at Ser616. In hepatocellular carcinoma, a high level of the minus-end MT protein CAMSAP2 correlates with Rac1/JNK activation, leading to both transcriptional repression of the tubulin deacetylase HDAC6 and consecutive high MT acetylation [78]. Also, in breast cancer, a high level of kinesin KIF15 correlates with a high JNK expression and bad patient prognosis [79].

Moreover, JNK activation can be mediated by MAPs. In *Drosophila*, the accumulation of active JNK/basket at embryonic motor axon tips depends on a Tau and spectraplakin/short stop (Shot)-mediated stabilization of MTs. At this location, as observed in mammals, the JNK/basket MAPK and its upstream MAPKKK activator DLK/Wallenda may phosphorylate cargoes to favor their unloading from the kinesin/unc104 motor [80]. Indeed, the depletion of both Tau and Shot results in abnormal accumulation of active P-JNK and kinesin cargoes in neuronal soma [80]. Note that the depletion of Shot only in epithelial cells is not sufficient to activate JNK, but instead activates DLK and the downstream MAPK p38 [81]. Similarly, depletions of the two plakins, plectin or desmoplakin, both being multi-cytoskeleton crosslinkers, also activate p38 and perturb the actin cytoskeletal morphology in mammalian cells [82,83].

Reversely, the disruption of the MT cytoskeleton can also result in the activation of JNK through the MAPKKK DLK to mediate axon regeneration [36]. Recently, destabilization of MTs has been shown to release the sequestered GEF-H1, triggering RhoA/MKK4-mediated JNK stimulation [84]. In parallel, a loss of centrosomes in *Drosophila* also stimulates JNK signaling pathway [85]. 

#### 2.2.2. Phosphorylation of Tubulin Proteins and MAPs by JNK

So far, tubulins have not been found as direct substrates of JNKs even if putative sites are present in beta and gamma tubulins (column 5 in Table S1). Instead, the kinases regulate MTs by phosphorylating a large set of MAPs. Generally, phosphorylation of neuronal structural MAPs by JNK stabilizes MTs by enhancing MAP affinity to sustain neuron development and function, and to protect MTs upon stress (for review [15,86,87]). Indeed, MAP1B phosphorylations at Ser25 and Ser1201 in rat are strongly associated with growing axons, but also with axon injury in transected nerves, and regenerating axons in sutured neurons [88]. MAP2 phosphorylations at Thr1619, Thr1622, and Thr1625 increase the dendritic complexity and stabilize MTs in rats [89]. Conversely, doublecortin phosphorylations by JNK1/2 at Thr321, Thr331, Ser334 (Thr326, Thr336, Ser339 in human) [90] and at Ser332 favor neurite development and neuronal migration by decreasing the affinity of this stabilizing MAP for tubulin and MTs [91]. Since doublecortin can switch from MT- to F-actin-binding [92], this may explain its positive effects on neurons [93] despite its release from MTs. Note that doublecortin contains a JBD [15] (Table S1) and all the other members of the doublecortin family (DCLK1/2, DCDC1/2, DCD2B, RP1/1L1) are able to bind the JIP JNK-scaffolding proteins and may therefore be substrates of JNK [94] (Section 5 on scaffolds). Also, the specific phosphorylations of doublecortin at Thr331 and Ser334 (in rat) can be removed by recruitment of the Ser/Thr phosphatase PP1γ1 by neurabin-II, an actin-binding protein enriched in migrating neurons [95]. The stabilizing MAP Tau is phosphorylated in vitro by JNKs at several sites, including Ser202, Thr205 and Ser422 [96], with misregulation occurring in Alzheimer’s disease [97] and epilepsy [98]. In the brain of Alzheimer patients, activated JNK is found in granules before symptom appearance and correlates with specific Tau conformational-misfolding known to prevent its binding to MTs [99]. At the beginning of the disease, phosphorylation at Ser422, located next to a caspase-mediated cleavage motif (D421), prevents Tau C-terminal cleavage in vitro, such that this truncated form only appears later [100]. However, during apoptosis induced by hyperosmolar stress, JNK stimulates caspase-3-dependent cleavage of Tau [101]. JNK can also mediate Tau decay by a BAG3-dependent autophagy mechanism in rat neurons [102]. Interestingly, a neuronal degenerative mouse model of synaptopathy, overexpressing human Tau that harbors a P301L mutation, revealed a sexual dimorphism, with a higher P-JNK level in female dendritic spines associated with a higher dementia level [103]. Last but not least, P-JNK and P-Tau (Ser202, Thr205) colocalize at mitochondrial sites with the non-fibrillar neurotoxic form of alpha-synuclein (Pα-syn), which is a truncated and phosphorylated synuclein found in Parkinson disease, that triggers mitochondrial damage [104]. The MT-destabilizing protein stathmin contains a JBD and is phosphorylated by JNK on Ser25 and Ser38 [105] (Supplementary Table S1). Such stathmin phosphorylations, recovered in stathmin-like proteins (SCG10, SCLIP), result in the case of SCG10 in the release of sequestered tubulin dimers, and lead to MT stabilization and dendritic growth of neurons [106]. Of note, JNK is also able to inhibit stathmin gene expression by increasing miR223 levels, a miRNA lost in malignant mesothelia [107]. Also, in conditions of osmotic stress, after heat shock or in the presence of arsenite, stathmin phosphorylation by JNK at Ser38 stabilizes MTs, allowing cytoprotection [108]. In a retro-control loop, phosphorylated stathmins are able to prevent JNK-mediated overactivation by oxidative stress in hepatocytes [109].

In addition to neuronal structural MAPs, JNK directly phosphorylates motors and cargoes to tightly regulate MT-based transport especially in neurons. Concerning the motor kinesin KIF5C, Ser176 phosphorylation by JNK3 facilitates peroxysome transport to MT plus-ends towards the cell periphery, but in stress conditions, like in Huntington’s disease, it can prime the motor to disengage entirely from MTs [110]. Concerning synaptotagmin-4 located on DCVs (dense core vesicles), its phosphorylation at Ser135 by JNK detaches the vesicles from the Kif1A kinesin motor leading to their capture at synapses by actin [111]. Also, phosphorylation of the β-amyloid precursor protein (APP) at Thr668 by JNK in Alzheimer disease [112] occurs in the domain necessary for the enhanced fast velocity (EFV) transport by kinesin-1 [113] but seems dispensable for this function [114]. A very similar site (Thr736) is phosphorylated by JNK in the APP-like protein APLP2 [115], with the synaptic adaptor Mint2/APBA2 being able to stimulate this phosphorylation. Consistently, phosphorylation of the cargo BimL (Bcl-2-like protein 11, a pro-apoptotic factor) at Thr56 by JNK, a site located in the binding-motif of dynein light chain DLC1, releases it from the dynein motor, thus triggering apoptosis [116]. A similar mechanism may occur for the pro-apoptotic factor Bmf, its phosphorylation by JNK at Ser74 mediating its release from DLC2/myosin V or DLC2/dynein motors [116]. Furthermore, phosphorylation at Ser421 of the adaptor JIP1 by JNK functions as a molecular switch to favor kinesin-1-mediated anterograde instead of dynein-mediated retrograde transport of APP vesicles [117]. The residues of the kinesin-1 light chain KLC1 required to bind JIP1 have been identified [118]. Note that JIP1 is also phosphorylated by JNK at several sites, including Thr103 and Thr205, which are required for its JNK-pathway scaffolding property [119] (Section 5). The related neuronal scaffolding and motor-associated protein JIP3/JSAP1 can also be phosphorylated by JNK at Thr266, Thr276, and Thr287 [120], and accumulates in the growth cone of neurites where it colocalizes with activated JNK and exocytic vesicles [121].

In epithelial cells, JNK1 and JNK2 can directly phosphorylate the MT-rescue factor CLIP-170 at Thr25, Thr45 and Ser147 with different kinetics and efficacies upon stress [122]. These phosphorylations located in serine-rich regions flank the CAP-Gly MT-binding and SxIP EB1-binding domains and allow frequent formation of short-lived CLIP-170 remnants at the trailing end of comets. As the transient CLIP-170 trailing spots overlap future rescue hotspots, this suggests that P-CLIP-170 functions more rapidly and more efficiently to prepare the MT lattice for future rescues. More indirectly, in G2 phase, JNK2 phosphorylates Ser274 of the Golgi protein GRASP65, preventing its capacity to stabilize Golgi-associated MTs [123]. JNK1 is also able to phosphorylate the mitotic spindle-associated MAP WDR62 at Thr1053 to prevent its binding along interphasic MTs [124]. This phosphorylation recruits the E3 ubiquitin ligase Fbw7 to mediate WDR62 proteasomal degradation [125]. Note that in the fly, P-JNK is found in intestinal stem cell (ISC) mitotic spindle MTs upon fasting or aging. There, it participates in the reorientation of the spindle, leading to symmetric division of ISC and to the loss of tissue homeostasis [126]. This relies both on a JNK-mediated WDR62 recruitment to spindles and on the transcriptional repression of the motor Kif1A. As mentioned later in this review (Section 5), WDR62 is also an effective scaffold protein in the JNK signaling pathway, stimulating JNK to initiate meiosis and to prevent infertility in mice [127].

Despite the characterization of numerous MAPs as substrates, a systematic search of docking sites for JNK suggested that more remained to be characterized, such as the dynein motor DYH12, the plus-end regulator APC2, the minus-end anchoring AKAP6 and the potential regulator of dynein FAM190A/CCSER1 [15,68,69]. Also, a phosphoproteomic analysis of purified growth cone samples revealed the presence of numerous SP/TP phosphorylated sites that could rely on JNK activity in several MAPs, including APC2 and several dynein-related proteins [70] (Section 6 and Supplementary Table S1). Similarly, both kinesins KIF5C and KIF5B/uKHC are found to be phosphorylated at S934 in mouse brain [128], a potential SP consensus site for JNK that has also been identified in the Kawasaki study [70]. As dynein and kinesin motors appear as preferred substrates for JNKs, it would be interesting to test if these regulations affect MT-dependent transport and organization of other cytoskeleton components such as actin, IFs, septins, and spectrins.

## 3. JNK and Non-Polarized Cytoskeletons: Intermediate Filaments and Septins

In contrast to actin and MTs, intermediate and septin filaments are nonpolar and do not behave as tracks for dedicated motors. They constitute two large families of proteins, of which the subunits and the organization into filaments vary depending on the cell type, especially for the IFs (Supplementary Table S1). Even if no polymerization/depolymerization occurs at the polymer ends, they can exchange internal segments of filaments and reorganize to adapt to cell life and environment (reviewed in [129,130]). IFs mostly protect the nucleus and link it to the plasma membrane and extracellular matrix via hemidesmosomes, desmosomes and focal adhesions. Septins can form filaments, bundles or rings and are often coaligned with the actin cytoskeleton. Mostly, septins function both as diffusion barriers (especially during septation in yeast, or at the base of the flagella and of dendrite spikes) or as scaffolds on actin filaments, MTs or membranous vesicles (for review [131,132]). Therefore, both intermediate filament proteins and septins associate with several partners, called IFAPs (for intermediate filament-associated proteins) and SeptAPs (for septin-associated proteins, here in the review), partly listed in Supplementary Table S1. So far, IFs seem to act as JNK stress sensors, and several variants found in diseases are associated with JNK overactivation. It appears that IF-constituting proteins and may be some IFAPs that are able to interlink other cytoskeletons, are phosphorylated by JNK (Supplementary Table S1). Concerning septins, the data related to JNK remain sparse, but the field might deserve more attention in the future. Indeed, SeptAPs might be essential to regulate JNK in the environment of F-actin or MTs.

### 3.1. JNK and Intermediate Filaments

IF proteins constitute a large family of 73 members (subtypes I to VII depending on the differentiated cell type, Supplementary Table S1) harboring a central rod domain implicated in coiled-coil dimerization, flanked by globular head and tail domains. Two dimers associate in an antiparallel fashion to build a symmetric tetramer, and 8 tetramers form the basic unit of the filament, a polymer of 10 nm diameter on average (for review [2,133,134]). Due to their structure, IFs are particular elastic components that deform rather than break upon mechanical stress. These abundant cytoskeleton proteins are often overexpressed during stress and accumulate post-translational modifications (PTMs) [129]. Indeed, the head and tail domains of IFs are highly regulated by phosphorylation by several kinases (for review [135]. 

#### 3.1.1. Control of JNK by IFs

Some IFs, such as desmin and vimentin (class III) or keratin 8 (K8, class II) are able to bind to JNK and modulate its activity. Desmin IFs are specifically localized to the skeletal muscle Z-disk, where they interconnect myofibrils laterally. In addition to a role in myofibrillar alignment, desmin is also implicated in JNK-mediated stress sensing, since it stimulates JNKs under stress [136]. Indeed, desmin, but also vimentin, both interact with JNK2 and the overexpression of one of them, as encountered in obstructed bladder smooth muscle (BSM) disease, triggers JNK activation, resulting in BSM lack of contractility [137]. By contrast, the binding of K8 to JNK sequesters the kinase, reducing its ability to phosphorylate its substrate c-Jun [138].

In different disease contexts, IF variants have been shown to induce JNK activation. Thus, acetaminophen (APAP)-mediated K8 hyperphosphorylation (both at Ser74 and Ser432 sites) in mice expressing human K8 variants (G62C or R341C sensitive to APAP injury, commonly found in European and North American populations) correlates with keratin network alteration and JNK activation [139]. Furthermore, the human K14 R125P variant (class I), found in the epidermolysis bullosa simplex (EBS) skin disease, is also known to trigger JNK activation [140]. As well, MLK2/JNK was found to be stimulated by the R239C variant of the glial fibrillary acidic protein (GFAP, class III) recovered in Alexander neurological disorder [141]. Altogether, these results highlight a general JNK activation property of IF variants found in pathologies.

#### 3.1.2. Phosphorylation of IF Proteins and IFAPs by JNK

JNKs are known to regulate different IF proteins, especially under stress conditions. For example, in epithelial cells, they directly phosphorylate soluble K8 (class II) at Ser74 in the head domain upon apoptotic Fas receptor (FasR) activation [138], and probably keratins K4-K5-K6 (class II), since they all harbor K8-related phophosites [142]. Note that phosphorylation at Ser74 in K8, which can also be mediated by p38 and Erk2 MAPKs, is abolished when the caspase-mediated cleavage sites are mutated in K18 [143] or in the G61C K8 mutant found in patients predisposed to liver injury [144]. In the latter case, K8 would not be phosphorylated anymore by MAPKs, resulting in the overphosphorylation of other non-keratin targets involved in apoptosis, leading to abnormal hepatocyte death under stress. In contrast, in skin of psoriasis patients and in squamous cell carcinomas (SCC) respectively, K6 and K5 phosphorylations increase at Ser74-related site [142]. K8 is also phosphorylated by JNK at Ser432, when the kinase binds to the transglutaminase-2 (TGase-2/TG2) upon sphingosylphosphorylcholine (SPC) lipid treatment, leading to keratin perinuclear reorganization and to enhanced cell migration [145]. This Ser432 phosphorylation might be relevant in cancer and metastasis formation since the SPC lipids are found in the malignant ascites of tumors [145]. 

In neurons, IFs (class IV) can be substrates for JNK3. Indeed, strong activation of JNK, upon arsenite or NaCl treatments, correlates with neurofilament heavy polypeptide (NFH) phosphorylations of multi-repeated “KSPxE” motifs [146], which are also present in neurofilament medium polypeptide (NFM) but not in neurofilament light polypeptide (NFL) filaments. These numerous “KSP” sites, located in the side arms projected away from the neurofilament axis, are also targets of other kinases including p38 and ERK1/2 MAPKs, making NFM/H one of the most extensive phosphoproteins characterized in axons [147,148,149]. These phosphorylations are normally present in dendrites, but upon stress-mediated JNK activation, they preferentially accumulate in the perinuclear regions of neurons [146,147], as encountered in amyotrophic lateral sclerosis [148]. Last but not least, Lamin B1, a nuclear IF (class V), is phosphorylated by JNK on Thr575 upon genotoxic stress (methylmethanesulfonate MMS), resulting in the release from the lamina of its binding partner, the Oct-1 transcription factor, which can then transactivate specific genes in order to execute a protective stress response [150]. 

This list might not be exhaustive yet, since GFAP (class III) has been found in a two-hybrid screen as a binding partner of JNKs, but in a JBD-independent manner [69]. Nestin (class IV) and peripherin (class III) might also be substrates of JNK in rat axonal growth cones [70] (Section 6 and Supplementary Table S1).

Even if a lot of IFAPs exist, so far to our knowledge, only the heat shock protein Hsp27, which limits keratin bundling in vitro [151], is indirectly phosphorylated at Ser78 (not SP site) upon JNK activation [152], but a possible role of this phosphorylation in IF network remodeling has not been tested yet. Otherwise, the interaction of the DNA-binding transcriptional repressor MOK2 with its lamin A/C-binding partner is prevented when MOK2 is phosphorylated at Ser38 and Ser129 by JNK3/JSAP1 [153]. Note that a large family of IFAPs, not reviewed in Supplementary Table S1, enclosed a hundred of KAPs (keratin-associated proteins) found in trichocyte cells, where they crosslink the keratin filaments in hair and wool fibers (for review [154]). Considering the importance of IFAPs in interlinking IFs to other cytoskeleton and cellular components, we speculate that some of these crosstalk proteins should be uncovered in the future as direct targets of JNKs. In Supplementary Table S1 are reported the number of SP/TP sites encountered in different IFAPs, including plakins and spectraplakins, that allow IF interaction with desmosomes, focal adhesions, MTs, actin filaments and mitochondria, but also in nesprin IFAPs that link IFs to the nucleus envelop, and in dedicated muscle IFAPs associated with sarcomeres [2,6,7,155]. Note that among IFAPs are also proteins that are known ABPs or MAPs: the formin FHOD3 (actin nucleator), the actin organizer filamin A (also related to septins and spectrins), some S100 proteins (related to actin and MTs), the FA-associated tensin-1 (related to actin), the postsynaptic-associated SAPAP1/GKAP/DLGAP1 (related to actin and MTs), the MT plus end-tracking protein (+TIP) APC (related also to actin), the MT motor KIF5B (related to spectrins) and the spectrin-associated ANK1 (related to MTs). Most of these proteins are highly enriched in SP/TP sites (Supplementary Table S1), are known JNK partners (Filamin A, S100, JBD in FHOD3) or were found phosphorylated on SP/TP sites in rat growth cones presenting high JNK activity (APC and KIF5B) [70] (Section 6 and Supplementary Table S1). One exciting possibility is that the phosphorylation of these multitask proteins by JNK could modulate several cytoskeletons at the same time, thereby revealing a key coordination role of JNK in global cytoskeleton regulation.

### 3.2. JNK and Septins

The septin family of GTPases is now considered as the fourth element of the cytoskeleton [3,156]. In humans, thirteen septin proteins have been identified that can be clustered in four classes according to their structure homology (Supplementary Table S1). Septins oligomerize by end-to-end binding of palindromic hexamers or octamers that can organize into filaments, bundles, gauzes or rings depending on the cell context. Septin filaments of 10 nm diameter in their pairwise organization are mostly found at the plasma membrane, along actin fibers, at the actomyosin contractile ring characteristic of the cleavage furrow, or along MTs in a few cell types or specific physiological or pathological conditions. For example, their MT localization greatly increases in response to the anticancer drug paclitaxel which contribute to confer cell resistance to the drug by restoring MT dynamicity [157,158]. 

So far, septins are not established substrates of JNK. However a close analysis of their sequences revealed the presence of SP/TP putative phosphorylation sites especially in classes 3, 7 and 2 compared to class 6 (Supplementary Table S1). Indeed, Kawasaki and colleagues [70] found by a phosphoproteomic screen that Ser334 of SEPT7 is phosphorylated in growth cones of rat neurons (Section 6 and Supplementary Table S1). Since JNK is activated at this location, it might trigger this phosphorylation. Reciprocally, the SEPT9_i1 isoform has been shown to bind to JNK, and its upregulation frequently recovered in cancers protects JNK from degradation, resulting in sustained kinase signaling [159]. 

At the molecular level, septins act mostly as scaffolds or diffusion barriers [132,160,161]. From the literature, the SeptAPs appear more as regulators of JNK than substrates. During vesicle fusion with targeted membrane, especially during exocytosis, septins associate with tethering complexes (the exocyst) and with fusion proteins (SNAREs), enabling their localized and timely interactions with vesicles [162]. The binding of the exocyst protein Sec8 to JIP4/JLP prevents this JNK-scaffolding protein to activate the downstream MKK4-JNK pathway, normally occurring during apoptosis [163]. Since SEPT9 mediates Sec8 recruitment to the midbody during cytokinesis [164], it might also regulate JNK at that location. Note that Sec8 might be a substrate of JNK in growth cones of rat neurons [70]. Also, the exocyst protein APBA2/Mint2 was found to contain a JBD domain [68]. Otherwise, three tSNAREs, syntaxin-1, syntaxin-2 and SNAP25 display a JBD domain (Supplementary Table S1), allowing their binding to JNK2 and JNK3 [165] to favor SNARE complex formation in the presynaptic compartment and subsequent neurotransmitter release [165]. Since septins are also enriched in presynaptic nerve terminals [166] and regulate SNARE complex function (for review [167,168]), we speculate that an interplay between septins, SNAREs and JNKs might occur in neurons. In the vesicular context, the SeptAPs synaptojanin, synapsin-2, VAPB and APBA1/Mint1 might be substrates of JNKs in growth cones [70] (Section 6 and Supplementary Table S1).

Meanwhile, the Cdc42 effector BORG3, considered both as a SeptAP and an ABP, mediates a SEPT9-dependent remodeling of F-actin in contractile stress fibers during cell migration [169]. Knowing that BORG3 is an inhibitor of JNK [170], and that the ABP anillin is a binding-partner of septins essential in the establishment of the actomyosin contractile ring for cytokinesis (for review [171]), a possible link between BORGs, septins and JNKs might be interesting to investigate in the context of actomyosin contraction. In the actin/septin field, filamin A, cofilin-1, and SLC9A3R2/NHERF2 might also be worth to look at as potential regulators or substrates of JNK [70,132,160,161].

Recently, septins have been screened as binding partners of the complex LRCH3/DOCK7/DOCK8/MYO6 on actin filaments [172,173,174]. DOCKs, corresponding to the Dedicator of CytoKinesis family, are GEF proteins of RhoA/Rac/Cdc42 that regulate F-actin organization (for review [175]) and MT dynamics [176]. In fly, DOCK3/sponge positively regulates JNK via Rac1 [177]. A similar regulation seems to occur in mammals through DOCK7 [178] and through DOCK180/DOCK1 or DOCK5 associated to the JNK-upstream regulator p130Cas ([176], for review [179] and Section 5). Interestingly, systematic search of JBDs suggested that DOCK5, DOCK7 are binding-partners of JNK [15,68]. 

In addition, a link between septins and JNK may exist in the environment of MTs. Indeed, septins are scaffold partners of MAPs on MTs, including the structural MAP4, the tubulin-deacetylase HDAC6 (for review [180]), the dynein/dynactin motor complex [181] and maybe the +TIPs CLIP-170 and MCAK [158]. Also, SEPT9 mediates a dynein-dependent retrograde transport of lysosomes, and this property is enhanced upon arsenite stress, a well-known JNK activator. Interestingly, MAP4, HDAC6 and dynein/dynactin, which are both MAPs and SeptAPs, all contain SP/TP sites which are phosphorylated in growth cones of rat neurons, suggesting that they may be substrates of JNK at that location [70] (Section 6 and Supplementary Table S1). CLIP-170 is also a known substrate of JNK regulating MT rescues [122]. Lastly, note that JIPs might be involved in septin transport, since endosomal trafficking of septin mRNAs, and their local translation and assembly in heteromeric complexes on MTs, is required in fungi to promote efficient septin cytoskeleton formation at appropriate location [182].

## 4. JNK and Newly Considered Cytoskeletons: Spectrin and ESCRT-III

Two types of protein polymers organized into filaments, spectrins and the endosomal sorting complex required for transport (ESCRT) are increasingly recognized as particular cytoskeletons, mostly associated with membranes. Here, we review their few known links with JNK. Since some of their associated proteins can be regulators of the previously mentioned cytoskeletons, we did not duplicate the protein names in Supplementary Table S1, but instead indicated them in red in the appropriate sections.

### 4.1. JNK and Spectrins

Originally identified in erythrocyte cells, spectrins are fibrous proteins associated with short actin filaments, which organize and stabilize specialized plasma membrane domains for appropriate cell architecture and elasticity. They are composed of two chain molecules (α and β, some called fodrins, Supplementary Table S1) that heterodimerize side-to-side in an antiparallel fashion to form a head-to-head tetramer (for review [8,9]). At the plasma membrane, they build a compact 2D-submembranous polygonal meshwork made of 6 tetramers, anchored to membranes in part through ankyrin proteins. Additionally, in neurons, they are part of a special membrane-associated periodic skeleton (MPS), organizing regularly spaced actin rings along axon and dendrite shafts. In that case, spectrin tetramers form stretched filaments running parallel to the cell projection axis. In all cells, spectrin tetramers are connected by junctional complexes, including short actin filaments, adducin, tropomodulin and protein 4.1. Like their common ancestor α-actinin, spectrins can regulate F-actin properties directly or through several ABPs. They can also regulate MT-mediated cargo transport, especially in neurons (for review [9,183,184,185]). Note that the extended spectrin family includes membranous regulators of F-actin (dystrophin, utrophin), but also of IFs (nesprins, plakins) and of MTs (spectraplakins) [8] (Supplementary Table S1), which are not discussed in this section.

To our knowledge, even if all spectrins present SP/TP sites (Supplementary Table S1), they are not characterized as substrates of JNK yet. However, the study of Kawasaki and colleagues [70] suggests that JNK may be able to phosphorylate four spectrins/fodrins in growth cones (Section 6 and Supplementary Table S1)**.** Consistently, JNK1/MAPK8 has been found in the spectrinome of βII-spectrin/SPTBN1/β-fodrin [186]. Reversely, spectrins seem to participate in the regulation of the JNK signaling. For example, in neurons stressed by trophic deprivation (TD), depletion of βII-spectrin/SPTBN1/β-fodrin protects axons from degeneration by inhibiting the retrograde signaling, such that trophic deprivation-activated DLK is unable to mediate JNK-dependent c-Jun phosphorylation [187]. In contrast, depletion in fly of neuronal α-spectrin triggers JNK/cFos-mediated degenerative synapse retraction at the neuromuscular junction (NMJ) [188]. 

Like spectrins, their close partners are not known as direct JNK substrates, even if their properties seem more or less directly regulated by the kinase. The expression of the constitutive MKK7D JNK-activator in heart inhibits the interaction between the αII-spectrin-SH3i isoform and the gap junction protein connexin-43, precluding its normal transport toward junctions [189]. The depletion of the scaffolding protein anillin also triggers a JNK-mediated disorganization of both adducin-spectrin and cortical actomyosin skeletons, as well as disruption of tight and adherence junctions (TJs, AJs) [190]. In that case, cotreatment with the SP600125 JNK-inhibitor is sufficient to reestablish the normal cytoskeletons and junctions. Note that among the common spectrin linker proteins, adducins, ankyrins and protein 4.1 are all enriched in SP/TP sites (Supplementary Table S1). Accordingly, phosphoproteomic analysis of purified growth cone samples revealed the presence of phosphorylated SP/TP sites, which could rely on JNK activity in both adducin and 4.1 proteins [70] (Section 6 and Supplementary Table S1). Also, several MT-associated motors known to bind spectrins were found phosphorylated in that study. Altogether, this points out spectrins and their partners as potential JNK-linked proteins to further study in the context of stress.

### 4.2. JNK and ESCRT-III

Inverse membrane budding (away the cytosol), observed, for example, during abscission, endosome maturation into multivesicular bodies, autophagy or plasma membrane repair is mediated by the “endosomal sorting complex required for transport” (ESCRT) protein machinery. ESCRT proteins assemble on the cytosolic face of the neck of the forming involution and cooperate with the ATPase VPS4 to drive membrane scission (for review [11,12]). During this process, ESCRT-III complex allows the formation of membrane-binding cytoskeleton spirals, made of the two ESCRT-III CHMP4 and CHMP2B proteins polymerized into filaments (for review [191,192]). 

Among the ESCRT-III proteins, some display putative target site(s) for JNKs, while most of the known interactors display at least one (Supplementary Table S1). However, data demonstrating effective phosphorylations are lacking so far. Mutations of CHMP2B causes front-to-temporal dementia (FTD), due to its failure to dissociate from the ESCRT complex. In this context, nervous system cells accumulate the scaffolding protein POSH/SH3RF1 implicated in JNK activation [193]. In fact, POSH/SH3RF1 is a binding partner of the ESCRT recruiter ALIX, and together with the mannosyltransferase ALG-2, they are able to activate JNK in fly [194]. Likewise, the depletion of the most downstream ESCRT component, the ATPase Vps4, leads to JNK signaling activation, apoptosis stimulation and tumorigenesis prevention in fly [195]. Reciprocally, the ESCRT-dependent exosome release is inhibited by JNK upregulation, leading to low levels of small extracellular vesicles secreted by astrocytes [196]. CHMP4B targeting to the abscission site at the latest step of cytokinesis is primed by sequential action of contractile machineries: first anillin and septins localize to three discrete rings within the intracellular bridge (ICB) to establish an initial ingression where MTs reorganize, then anillin dissipates and septins recruit ESCRT-III proteins at the final constriction site [197]. There, ESCRT-III filament might form a helix that will dissipate later on and that is involved in spastin recruitment for MT severing at the constriction site [198]. It will be interesting to see if JNK could participate in the orchestration of all these cytoskeleton dynamics or interfere with it in conditions of stress.

## 5. Cytoskeletal Localization of Upstream JNK Regulators and Scaffolds 

JNK phosphoactivation (on the TPY motif) by different stresses and developmental conditions is mediated by the MAPK upstream pathways. This process is generally stimulated by scaffolding proteins, like JIPs, WDR62, β–arrestins and filamins that tether the components of the pathway, but also by adaptor proteins reviewed below. Reversely, the magnitude and duration of signal transduction is attenuated by DUSP-mediated dephosphorylation of the TPY site. Hereafter, we review special cytoskeletal localizations of such upstream JNK regulators.

As previously discussed, JIPs are important scaffolds of the JNK pathway [18] and well-known cargoes and regulators of kinesin motors on MTs (for review [15,199]). Interestingly, JIP2 decorates cytoplasmic filaments in insulinoma cells, while JIP1 accumulates at their distal tip [200]. JIP1, JIP2 and JIP3/JSAP1 can all accumulate at axonal growth cones [75]. JIP4/JLP codistributes with perinuclear vesicles which are trafficking via MTs from endosomes to the TGN compartment [201], while JIP3/JSAP1 colocalizes with P-JNK at exocytic vesicles [121]. Like JIP3/JSAP1, JIP4/JLP can also colocalize with Tau-decorated MTs in neurons [202]. Similar to JIPs, when expressed in fibroblasts, the MAPKKK MLK2 colocalizes with activated JNK to punctate structures along MTs with the motor KIF3A [203]. 

WDR62 is also a scaffolding protein in the JNK signaling pathway, co-immunoprecipating with both MAPKKs and MAPKKKs ([204,205,206,207], for review [208]). WDR62 localizes at the spindle poles during mitosis [124] and can be recruited on spindles by JNK in intestinal stem cells [126]. It also localizes to stress granules upon arsenite stress [209]. 

Concerning the scaffold protein β-arrestin-2/3 of the JNK3 pathway (reviewed in [14,17,210]), it can decorate MTs in HEK293 cells in order to sequester/recruit proteins ([211], reviewed in [212]). It also colocalizes with JIP3/JSAP1 in a filamentous perinuclear pattern in Cos7 cells [213]. Given the recently established β-arrestinome [214], we can speculate that β-arrestin-2 also associates with or regulates F-actin, ABPs and the IF vimentin. 

The ABP filamins can also be JNK scaffolds (reviewed in [16,30]). Filamin A localizes with the MAPKK MKK7 on F-actin fibers in HeLa cells and this cytoskeletal binding enhances sorbitol-mediated JNK activation [28]. However, an influenza A virus nucleoprotein (NP) has recently been shown to sequester filamin A to favor viral replication by activating the cellular TRAF2/ASK1/JNK pathway [215]. By binding to the kinases MEKK1/MKK4/JNK1, filamin B is a scaffold for the JNK-pathway activation during membrane ruffle formation upon type I interferon treatment [29]. Interestingly, upon mechanical stress, filamins can stretch and extend to recruit actin at the cortex (for review [216]).

At focal adhesions, the versatile scaffold protein p130cas bound to Src is also a protein that can stretch upon mechanical stress. In that case, it is retrieved from FAs by actin inward flux and associates with the adaptor Crk to become a JNK-pathway activating factor via DOCK1/180 and Rac stimulation ([217], for review [216,218]. Also, p130cas associates with vimentin when this IF protein is not phosphorylated on S56 (SP site), precluding p130cas-mediated actin polymerization in relaxed muscle [219]. Reversely, muscle stimulation correlating with higher P-JNK levels and recruitment of Src to dystrophin [220], releases p130cas from vimentin and allows actomyosin contraction [219]. The p130cas-Crk complex can also recruit the ESCRT proteins through Tsg101 during the KHSV herpesvirus infection, allowing post-macropinocytic trafficking of the virus [221]. 

The versatile adaptor Rack1 is also considered as a MAPKKKs/MKK7/JNK scaffolding protein in human hepatocellular carcinoma [222]. In *C. elegans*, Rack1 directly binds to the p50/dynamitin protein in the dynactin complex [223]. Rack1 can also directly bind to β-actin [224] and α-spectrin [225]. 

Among the dual phosphatases, which are usually JNK inhibitors, but which can also have a scaffold role in the pathway (Supplementary Table S1), DUSP8 partly colocalizes with MTs [226]. Also, according to the human protein atlas (https://www.proteinatlas.org/, accessed on 4 August 2021), DUSP6 forms uncharacterized cytoplasmic filaments, while DUSP18 seems to localize at centrosomes.

## 6. JNK and Cytoskeleton Regulations in Time and Space: The Technical Challenge of Looking for Needles in a Haystack

From this overview, it appears that the cytoskeleton-JNK interrelationships are numerous and that the multifunctional JNK kinases orchestrate the organization, dynamic and function of these different types of filaments. As JNK-mediated phosphorylations are likely to occur locally and transiently on cytoskeletal elements, they can be easily missed by Western blot or mass spectrometry analysis of cells or tissues. Also, microscopic evaluation of the delimited and short-lived effects on the cytoskeleton can be a hard task, especially considering the complex interplays between the various kinds of filaments and the difficulty to specifically inhibit JNK isoforms at the right place and the right time. Here, is described a panel of technical approaches available to find new substrates of JNK and to decipher their cytoskeleton-related functions. 

A first available approach to identify new substrates, already presented along this review, consists in purifying cell subsets to study the phosphoproteome, for example, by using only growth cone membranes (GCM) of neurons. Indeed, systematic analysis of phosphoproteins in GCM of postnatal rat forebrains revealed that encountered phosphosites in that structure were highly proline directed and mostly under the control of JNK [70]. In this study, MTs and cortical cytoskeleton-related proteins were specifically enriched. Concerning MTs, no SP/TP sites were found phosphorylated in tubulin isoforms, while recovered in more than 35 MT-related proteins, including structural MAPs, +TIPs and molecular motors (column 9 in Supplementary Table S1). Like for tubulin, actin proteins in the growth cone did not exhibit phosphorylated SP/TP sites, which were however found in at least 23 ABPs involved in F-actin assembly and disassembly, as well as in motor activity. For the other cytoskeletons, Kawasaki and colleagues [70] found several phosphorylated SP/TP sites in neurofilaments (NFH, NFM3), nestin, peripherin, spectrins and associated proteins, septAPs, SEPT7 but none in ESCRTs. 

An alternative approach to identify new cytoskeletal substrates could rely on the use of a JNK kinase modified in the ATP pocket, which would be designed to accommodate a bulky labeled ATP analog for “tagging” the phosphorylated substrates in order to allow their subsequent purification and proteomic analysis [227,228]. Also, a computational approach could be used to predict MAPK docking site-containing proteins, to further experimentally validate the cytoskeletal JNK-interactome [68]. Finally, a refined yeast two-hybrid analysis using alternative JNK isoforms as baits could help to decipher common and specific partners as well as docking site properties in the context of cell cytoskeleton [69].

Furthermore, technology is now available to monitor kinase activity in space and time in live cells using FRET-based biomolecular kinase activity reporters (called BimKARs and more precisely JNKAR for JNK) [229]. For that purpose, a fluorescent protein containing the TP and the JNK docking sites of JDP2 (Jun dimerization protein 2) can generate a FRET signal upon phosphorylation by recruiting a fluorescent phospho-threonine binding domain of FHA1. With this JNKAR transgene, JNK activation upon cell exposure to anisomycin could be followed in real time. Recently, a related FRET-sensor approach was coupled to local optogenetic inhibition of JNK [230]. In that case, a mRuby2-JNKAR1EV-clover sensor was used in hippocampal neurons to visualize the effect of a photoactivatable JNK inhibitor (LOV2-JBD). By monitoring mCherry-actin in real time, the authors showed that JNK local inhibition in dendritic spine heads drastically decreases, in a few seconds, the motility of actin. Last but not least, the Duolink™ Proximity Ligation Assay (PLA) has been successfully used to demonstrate phospho-JNK dot-like association with F-actin as soon as 5min after a shear stress [33].

Also, it would be interesting to systematically look at the localization of proteins that can regulate JNK signaling in space and time, such as JNK-activating scaffolding proteins, or JNK inhibitory dual phosphatases DUSPs (Supplementary Table S1 and Section 5). Of course, the use of high-resolution microscopy (STED) and sample expansion to visualize the cytoskeleton and JNK could be a great step forward in the field [231,232,233,234]. 

The last issue we want to tackle here is the challenge for designing specific and efficient inhibitors of each of the 16 JNK isoforms, usable in therapy without cytotoxicity, for example to treat neuropathies or cancer (for review [199]. In basic research, the commonly used ATP-competitive SP600125 inhibitor has the disadvantages to inhibit all JNKs, to even inhibit, if too concentrated, other unrelated kinases (for review [235]) and to present side effects if used in therapy. DARPin libraries, corresponding to “Designed Ankyrin Repeat Proteins”, contain stable small proteins and have been used to select a specific inhibitor of the JNK1α1 form [236]. Also, the antitumor molecule Reversine specifically inhibits JNK1, but not JNK2 [237]. As previously discussed, optogenetic inhibition of JNK [230] allows transient and local stimulation of a JNK-binding domain (JBD) acting as a dominant negative inhibitor, by just photostimulating cells a few seconds at 458 nm (blue light). Finally, one recent study pointed out previously FDA-approved drugs as possible useful JNK3 inhibitors (dabigastran, estazolam, and pitavastatin) to reverse side effects of cerebral ischemia in rat [238]. Also, administration of the glycine amino acid in mice seems as efficient as SP600125 to prevent JNK phosphorylation and to confer neuroprotection [239].

## 7. Concluding Remarks

The cytoskeleton is made of several structures and is regulated by a huge amount of protein crosstalks, allowing cell adaptation to the environment and life cycle. In this context, JNK controls several steps that are most likely restricted in space and time, while in turn its activity is also specifically regulated by the cytoskeleton. In the future, a thorough utilization of sequence databanks, proteomic approaches, high-resolution microscopies should reveal new substrates of JNK. Also, since cytoskeletons are rather dynamic structures, we believe that video imaging of live cells would bring new information in the field of JNK localization and function. Lastly, the interplay between the different type of cytoskeleton filaments upon JNK-mediated stress and development should be explored more precisely. Considering that JNKs and cytoskeletons are misregulated in diverse diseases, including cancers and neuropathies, expanding the knowledge on their interplay should bring new important information for applied medical research in the future.

## Data Availability

Not applicable.

## Supplementary Materials

The following are available online at https://www.mdpi.com/article/10.3390/ijms22168375/s1, Table S1: Human cytoskeleton proteins and partners.
