# Supplementary material for: Cytoskeleton and Associated Proteins: Pleiotropic JNK Substrates and Regulators
Source: Int J Mol Sci. 2021 Aug 4;22(16):8375. doi: 10.3390/ijms22168375 (PMC8395060; doi:10.3390/ijms22168375)
Supplement: Supplementary file 1 [file ijms-22-08375-s001.zip › ijms-1303806-supplementary.pdf]

Supplementary Table S1 Human cytoskeleton proteins and partners.

1	2	3	4	5	6	7	8	9
**Cytoskeletons**	**Categories/Functions**	**Protein Names**	**Abbreviations**	**SP/TP Sites**	**Total Amino Acids in Proteins**	**Known JNK Substrates**	**JNK Binding–Known JBDs**	**SP/TP Sites Phosphorylated in Rat Growth Cones** [70]
**Actin**		actin cytoplasmic 1 (spectrin)	ACTB/ACT5	1	375	TAASSSS (S232) indirect *C. elegans* [43]		
		actin cytoplasmic 2	ACTG	1	375			no site
		actin muscle	ACTH, ACTA, ACTS, ACTC	0	376, 377, 377, 377			
						F-actin activates JNK [33]. F-actin disruption activates DLK/JNK [36]		
**Tubulin**	alpha	tubulin alpha TUBA1A, 1B	TUBA1A, 1B	0	451			no site
		tubulin alpha TUBA1C, 3C, 3D, 3E, 4A, 8	TUBA1C, 3C, 3D, 3E, 4A, 8	0	448-451			
	beta	tubulin beta TUBB2A, 6, 8	TUBB2A, 6, 8	2	444-450			
		tubulin beta TUBB3	TUBB3	2	450			no site
		tubulin beta TUBB4A, 4B	TUBB4A, B	2	444, 445			no site
		tubulin beta TUBB5	TUBB5	2	444			no site
	gamma	tubulin gamma	TUBG1	5 (KSP)	451			
						MT disruption activates DLK/JNK [36]		
**Intermediate filament**	I (simple epithelial, epiderm) acidic	keratin 10, 17, 18, 19, 24, 25, 27	KRT10, 17, 18, 19, 24, 25, 27	0	584, 438, 430, 400, 525, 450, 459			
		keratin 9, 14, 16, 20, 26, 28	KRT9, 14, 16, 20, 26, 28	1	623, 472, 473, 424, 468, 464	K14 R125P activates JNK [140]		
		keratin 12, 15	KRT12, KRT15	2	494, 456			
		keratin 13	KRT13	4	458			
		keratin 23	KRT23	5	422			
	II (simple epithelial, epiderm)	keratin 1, 2	KRT1, KRT2	0	644, 639			
		keratin 3, 7	KRT3, KRT7	1	628, 469			
		keratin 4, 6	KRT4, KRT6	1	520, 564	(SLL**TP**LHV KRT4, SLL**TP**LNL KRT6)? [142]		
		keratin 5	KRT5	2	590	(SLL**TP**LNL)? [142]		

		keratin 8	KRT8	2	489	SLL**SP**LVL GLT**SP**GLS (S74, S432) [139,145]. Sequesters JNK [138]		
	III (mesenchym, muscle, astrocyte)	vimentin (spectrin)	VIM	1	466	activates JNK in bladder disease [136]	JNK2 binding [137]	
		glial fibrillary acidic protein	GFAP	0	432	GFAP R239C activates JNK [141]	JNK binding (indpt of JBD) [69]	
		desmin (spectrin)	DES/DES	4	470	activates JNK in bladder disease [136]	JNK2 binding [137]	
		peripherin	PERI/PRPH	3	470			1 site GSG**SP**SSS (but GSA**SP**SSS in human)
		syncoilin	SYNCI/SYNC	4	482			
	IV (nervous system)	nestin	NEST/NES	26 (KSP)	1621			7 sites PHG**SP**VR ELM**SP**K FPR**SP**EEE SLR**SP**EEE SLR**SP**EEE QRL**SP**QGD LVA**SP**VHL (not conserved in human)
		alpha-internexin	AINX/Ina	0	499			no site
		synemin	SYNEM/SYNM	16	1565			
		neurofilament light polypeptide	NEFL/NFL	1	576			no site
		neurofilament medium polypeptide	NEFM/NFM	17 (KSP)	916	(K**SP**xE repeats)		8 sites EK**SP**VK**SP**E LDV**SP**AEE AKE**SP**KEE GDK**SP**QES (VSG**SP**SSG AK**SP**VPK**SP**VE also in human)
		neurofilament heavy polypeptide	NEFH/NFH	48 (KSP KTP)	1026	K**SP**xE repeats [146]		>9 sites GTS**SP**DLP SSE**SP**SDP GNE**TP**LGQ KQE**SP**VEE DPE**SP**EGQ DDV**SP**EEQ LRP**SP**EAI AEG**SP**ETE (repetitions EAK**SP**AEA also in human)
	V (nucleus)	prelamin-A/C (actin, spectrin)	LMNA	10	664			
		lamin-B1	LMNB1	5	586	QQG**TP**RAS (T575) [150]		
		lamin-B2	LMNB2	9	620			
	VI (lens)	filensin	BSFP1	8	665			
		phakinin	BSFP2	1	415			
		paranemin (muscle Gallus)	paranemin	18	1748			
**Septin**	2	septin 1	SEPT1	2	367			

		septin 2	SEPT2	3	361			
		septin 4, 5	SEPT4, SEPT5	5	478, 369			
	3	septin 3	SEPT3	4 (KTP)	358			
		septin 9 isoforms (actin MT)	SEPT9	9 (KTP)	586	SEPT9-i1 prevents JNK degradation [159]		
		septin 12	SEPT12	8	358			
	6	septin 6, 11	SEPT6, SEPT11	0	434, 429			
		septin 8, 10, 14	SEPT8, SEPT10, SEPT14	1	483, 454, 432			
	7	septin 7	SEPT7	4 (KSP)	437			1 site LTK**SP**LAQ (conserved in human)
**ESCRT-III**	core	charged multivesicular body protein 2a/ Vps2A	CHMP2A	1 (KTP)	222			
		charged multivesicular body protein 2b/ Vps2B	CHMP2B	0	213			
		charged multivesicular body protein 3/ Vps24	CHMP3	0	222			
		charged multivesicular body protein 4a, b, c/(Snf7) (anilin, septin)	CHMP4A, CHMP4B, CHMP4C	1	222, 244, 233			
		charged multivesicular body protein 6/ Vps20	CHMP6	0	201			
	peripheric	charged multivesicular body protein 1a, b/Did2A (nuclear envelope, MT spastin)	CHMP1A, B	1	196, 199			
		charged multivesicular body protein 5/ Vps60	CHMP5	1	219			
		charged multivesicular body protein 7/ Did2 (nuclear envelope)	CHMP7	4 (KSP)	453			
	diassembly	vacuolar protein sorting-associated protein IST1 (nuclear envelope, MT spastin)	IST1	2	364			
**Spectrin**	non erythrocytic	spectrin alpha chain, non-erythrocytic 1/ a-fodrin/αII-spectrin (MT kinesin, lamin)	SPTN1/SPTAN1	3 (KSP)	2472	if depleted: activates JNK (*Drosophila*) [188]		1 site QEK**SP**REV (conserved in human)
		spectrin beta chain, non-erythrocytic 1a/ β-fodrin/βII-spectrin (actin, MT dynactin kinesin)	SPTB2/SPTBN1	8	2364	if depleted: TD-activated DLK is unable to activate JNK [187]	JNK1 binding? [186]	6 sites RPP**SP**EPS EQG**SP**R ES**SP**VP**SP**TS TLE**TP**AAQ SES**SP**GKR (but ES**SP**IP**SP**TS TQE**TP**SAQ in human)
		spectrin beta chain, non-erythrocytic 2/ βIII-spectrin (neuron) (actin, MT dynactin)	SPTN2/SPTBN2	6	2390			no site

		spectrin beta chain, non-erythrocytic 4/ βIV-spectrin (neuron) (actin)	SPTN4/SPTBN4	8	2564			1 site VAG**SP**ELG (conserved in human)
		spectrin beta chain, non-erythrocytic 5/ βV-spectrin (photoreceptor) (actin, MT dynactin kinesin)	SPTN5/SPTBN5	13	3674			
	erythrocytic	spectrin alpha chain, erythrocytic 1/ αI spectrin	SPTA1	6 (KTP)	2419			
		spectrin beta chain, erythrocytic/ βI-spectrin (actin)	SPTB1	6	2137			1 site ERQ**TP**DRP (not conserved in human)
**ABP/actin**	polymerization (+/− nucleation, bundling, capping, severing)	formin-1 (MT)	FMN1	18 (KTP)	1419		**R**SE**L**Y**L**D**L** (NFAT4-like) [15,68]	
		formin-2	FMN2	19 (KTP)	1722			1 site IQT**SP**TEE (conserved in human)
		formin-like protein 1	FMNL1	11 (KSP)	1100			
		formin-like protein 2	FMNL2	7 (KSP)	1086			
		formin-like protein 3	FMNL3	4 (KSP)	1028			
		FH2 domain-containing protein 1 (MT)	INF1/FHDC1	(KTP KSP)	1143			
		inverted formin (MT)	INF2	9	1249			
		FH1/FH2 domain-containing protein 1 (MT) (SEPT7/ROCK2)	FHOD1	12	1164			
		FH1/FH2 domain-containing protein 3 (nestin IF)	FHOD3	14 (KSP)	1422		**R**AD**L**S**L**D**L** (NFAT4-like) [15,68]	
		disheveled-associated activator of morphogenesis 1 (MT)	DAAM1	2	1078			
		disheveled-associated activator of morphogenesis 2 (MT)	DAAM2	3	1068			
		protein diaphanous homolog 1 (MT)	DIAPH1, mDia1	7	1272			
		protein diaphanous homolog 2	DIAPH2, mDia2	2	1101			
		protein diaphanous homolog 3	DIAPH3, mDia3	7	1192			
		delphilin (formin family)	GRD2I/GRID21P	19	1211			
	nucleation	actin-related protein 2 (branched)	ARP2	0	394			
		actin-related protein 3, 3B (branched)	ARP3, ARP3B	2	418, 418			
		spire homolog 1 (mb) (MT)	SPIR1	9	756	p150-Spir (phosphorylated by JNK in *Drosophila*) [54]	KQKRSSARNRTI QN**L**T**L**D [54]	2 sites QPS**SP**GGE (RPV**SP**EEI also in human)
		protein cordon-bleu	COBL	16 (KSP)	1261			
		vasodilator-stimulated phosphoprotein (spectrin)	VASP	5 (KTP)	380	RRV**S**NAG, RKV**S**KQE (S157		

					S239) indirect (PKA/PKG) [57]	
Arp2/3 regulation	Wiskott–Aldrich syndrome protein family member 1 (spectrin)	WASF1/WAVE1	10	559		
	Wiskott–Aldrich syndrome protein family member 2	WASF2/WAVE2	9	498		
	Wiskott–Aldrich syndrome protein family member 3	WASF3/WAVE3	6	502		
	Wiskott–Aldrich syndrome protein (spectrin)	WASP	6 (KTP)	502		
	neural Wiskott–Aldrich syndrome protein	WASL	5	505		
	protein BRICK1	BRK1/BRICK1	0	75		
	Nck-associated protein 1	NCKP1/NCKAP1/ NAP1	4	1128		
	abelson interactor 1 (ENAH phosphorylation via Abl) (spetrin)	ABI1	11	508		1 site KPP**SP**PVS (but KPP**SP**PMS in human)
	abelson interactor 2 (ENAH phosphorylation via Abl) (MT)	ABI2	12	513		
	WAS/WASL-interacting protein family member 1	WIPF1/WIP	17	503	overexpression activates JNK [32]	
	proline-serine-threonine phosphatase-interacting protein 1 (septin?)	PSTPIP1	6	416		
	Src substrate cortactin (possible linker for tight junction, adherens junction, dynamin, shank, septin)	SRC8/CTTN	3	550		
capping (pointed-end)	tropomodulin-1, 2, 3 (spectrin)	TMOD1, TMOD2, TMOD3	0	359, 351, 352		
	tropomodulin-4 (spectrin)	TMOD4	4 (KSP)	345		
	leiomodin-1	LMOD1	9 (KTP)	600		
	leiomodin-2	LMOD2	10 (KTP)	547		
	leiomodin-3	LMOD3	1	560		
capping (barbed-end)	Gelsolin	GELS	5 (KTP)	782		
	F-actin-capping protein subunit alpha-1, 2 (spectrin)	CAPZA1, CAPZA2	2	286, 286	if JNK phosphorylates CapZIP S108 = dissociation [47]	
	F-actin-capping protein subunit alpha-3 (spectrin)	CAPZA3	0	299		

	F-actin-capping protein subunit beta (spectrin)	CAPZB	2	277		
	CAPZ interacting protein	CapZIP/RCSD1/ CPZIP	18 (KSP)	416	ASPK**SP**GLK (S108) (GEEK**SP**PNA VKS**SP**LIE APG**SP**LSS) [47]	
inhibition of capping	F-actin-uncapping protein LRRC16A	CARL1/CARMIL1	21 (KTP)	1371		
	capping protein, Arp2/3 and myosin-I linker protein 2	CARL2/CARMIL2	22	1435		
	capping protein inhibiting regulator of actin dynamics	CRACD	17 (KTP)	1233		
depolymerization (+/- debranching)	cofilin-1 (septin)	COF-1	1	166	siRNA activates JNK [31]	
	cofilin-2	COF-2	0	166		
	cofilin non-muscle	E9PK25/CFL1	3	204	MVSPGHG (S3) indirect (LIMK/SHH/Omi/CAMKII) or JNK? [51,52]	2 sites SLG**SP**SAS (KSS**TP**EEV also in human)
	destrin	DEST/DSTN	1	165	siRNA activates JNK [31]	
	twinfilin-1	TWF1	1	350		
	twinfilin-2	TWF2	2	349		
	drebrin	DREB/DBN1	9	649		1 site PTR**SP**SD (conserved in human)
	drebrin-like protein/HIP55 (inhibit debranching) (spectrin)	DBNL	5 (KSP)	430		
	glia maturation factor beta, gamma (Arp2/3 inhibition)	GMFB, GMFG	2	142, 142		
	coactosin-like protein	COTL1/CLP	0	142		no site
	adenylyl cyclase-associated protein 1	CAP1	3	475		
	adenylyl cyclase-associated protein 2	CAP2	5	477		
	[F-actin]-monooxygenase MICAL1	MICAL1	14 (KSP)	1067		
	[F-actin]-monooxygenase MICAL2	MICAL2	15 (KSP)	1124		
	[F-actin]-monooxygenase MICAL3	MICAL3	35 (KSP)	2002		no site
G-actin binding	thymosin beta-4, 10, 15A, 15B	TYB4, TY10, TY15A, TY15B	0	44, 44, 45, 45		
F-actin dynamic	profilin-1	PROF1	2	140		
	profilin-2	PROF2	1	140		
	coronin-1A, 1C	COR1A, COR1C	1	461, 474		
	coronin-1B	COR1B	2	489		
	coronin-2B	COR2B	4	480		

	coronin-7	CORO7	13	925			
	smoothelin-like protein 2	SMTL2/SMTNL2	14	461	AAL**SP**MSA, EVI**TP**W**TPSP**SEK (S217 T236 T239 S241) [45]	PPRP**RP**VS**L**S**L**R LP (JIP-like) [45]	
crosslinking	alpha-actinin-1, 2 (spectrin)	ACTN1, ACTN2	2	892, 894		JNK binding, MEKK1 binding [26,27]	
bundling	alpha-actinin-3 (spectrin)	ACTN3	3	901			
	alpha-actinin-4 (spectrin tight juntion MICALL2?, septin)	ACTN4	1	911			
	transgelin	TAGL/TAGLN	0	201			
	advillin	AVIL	4 (KSP)	819			
	plastin-1, 3	PLS1, PLST/PLS3	3	629, 630			
	plastin-2	PLSL/LCP1	2	627			
	villin-1 (microvilli)	VIL1	6	827			
	homer protein homolog 1	HOMER1/HOM1	5	354			
	homer protein homolog 2	HOMER2/HOM2	2	354			
	homer protein homolog 3	HOMER3/HOM3	4	361			
	anillin (septin)	ANLN	24 (KTP)	1124			
	MARCKS-related protein	MRP/MARCKSL1	8	195	SAS**SP**TEE AAA**TP**ESQ EPS**TP**SGP (S120 T148 T183) [44]		
	myristoylated alanine-rich C-kinase substrate	MARCKS	10	332			2 sites GDA**SP**AAA EPG**SP** (conserved in human)
parallel bundling	fascin-1	FSCN1	2	493			
	fascin-2	FSCN2	1	492			
	fascin-3	FSCN3	3	498			
crosslinking to membrane	filamin-A (vimentin IF, spectrin, septin)	FLNA	39 (KSP)	2647	activates MKK7/JNK [16,28]	JNK scaffold [28]	
	filamin-B	FLNB	42 (KSP)	2602	activates MKK4/JNK [16,28,29,30]	JNK scaffold [28,29,30]	
	filamin-C	FLNC	37 (KSP)	2725	activates JNK [16,28]	JNK binding [28]	
	dematin/erythrocyte membrane protein band 4.9 (bundling) (spectrin)	DEMA/DMTN/ EBP49	13	405			
	ezrin (spectrin)	EZRI/EZR	1	586	ezrin Y353 binds MKK7 and activates JNK on endosomes [41], MEKK1/MKK4-7/JNK [42]		

	radixin (spectrin)	RADI/RDX	1	583	activates MEKK1/MKK4-7/JNK [42]		
	moesin (spectrin)	MOES/MSN	3	577	activates MEKK1/MKK4-7/JNK [42]. KYK**T**LRQ (T558) indirect [58]		
	Na+/H+ exchanger regulatory factor NHE-RF1, ERM binding protein 50	NHERF1/NHRF1/ EBP50/SLC9A3R1	4	358			2 sites QD**SP** E**SP**R (conserved in human)
	Na(+)/H(+) exchange regulatory cofactor NHE-RF2 (septin)	SLC9A3R2/NHERF 2/NHRF2	6	337			1 site LHL**SP**TAA (conserved in human)
	Ena/VASP-like protein (MT kinesin, spectrin)	EVL	9 (KSP)	416			
	protein enabled homolog (spectrin)	ENAH	0	591			
	lamellipodin (ENA/VASP binding) Ras-associated and pleckstrin homology domains-containing protein 1	LPD/RAPH1	26	1250			
	supervillin	SVIL	26 (KTP)	2214			
	cd2-associated protein/cas ligand with multiple SH3 domains	CD2AP	7	639			
	alpha-1-syntrophin/59 kDa dystrophin-associated protein A1 acidic component 1	SNTA1/59-DAP	7	505			
	beta-1-syntrophin, beta-2-syntrophin	SNTB1, SNTB2	9, 9	538, 540			
	dystrobrevin-alpha (MT kinesin)	DTNA	9 (KSP)	743			2 sites LVP**SP**TSE S**SP**SHT (conserved in human)
	dystrobrevin-beta (MT kinesin)	DTNB	7 (KSP)	627			
	SH3 and multiple ankyrin repeat domains protein 1 (fodrin)	SHANK1	57	2161		**RP**SS**L**P**I** (JIP-like) [15,69]	2 sites FQE**SP**K **SP**TSP (conserved in human)
	SH3 and multiple ankyrin repeat domains protein 2/cortactin-binding protein 1	SHANK2/CortBP1	32 (KSP)	1470			
	SH3 and multiple ankyrin repeat domains protein 3 (fodrin)	SHANK3	42 (KSP)	1731		JNK3 binding [56]	
	disks large homolog 4/postsynaptic density protein 95 (MT)	DLG4/PSD95/ SAP90	5	724	RRY**SP**VAK (S295) [55]	JNK3 binding [56]	
	disks large-associated protein 1/guanylate kinase-associated protein (DLG4 binding) (IF, MT)	DLGAP1/DLGP1/ SAPAP1/DAP1/ GKAP	12 (KTP)	977			
	disks large-associated protein 2 (DLG4 binding)	DLGAP2/DLGP2/ SAPAP2/DAP2	16 (KTP)	1054			no site

	dystrophin (MT) (spectrin family)	DMD	23 (KTP)	3685			3 sites PAS**SP**QLS NQD**SP**LSQ TVS**SP**STS (conserved in human)
	utrophin/dystrophin-related protein-1 (spectrin family)	UTRN/UTRO/ DRP-1	16 (KSP)	3433			no site
focal adhesion/adherence junction	talin-1 (spectrin, MT)	TLN1	12	2541			
	talin-2	TLN2	10	2542			1 site DEG**TP**PEP (conserved in human)
	vinculin (tension) (spectrin)	VINC/VCL	8 (KTP)	1134			
	tensin-1 (plectin p1c p1f/vimentin ?)	TENS1/TNS1	50 (KSP KTP)	1735			
	tensin-2	TNS2	38	1409			
	tensin-3	TENS3	33	1445			
	tensin-4	TENS4/TNS4	20	715			
	zyxin (tension)	ZYX	11 (KSP)	572			
	paxillin (tension) (spectrin, MT)	PAXI	13	591	GAL**SP**LYG (S178) [60]. MLK3, MEKK2, TG2/DLK dependent? [61–64]		
	focal adhesion kinase (MT)	FAK	8	1052	p130Cas/Rac1/MKK4 activates JNK [65]		
	afadin	AFAD/AFDN/AF6	22 (KTP)	1824			5 sites VEL**SP**GRR PDI**SP**TER NQP**SP**MMQ QPP**SP**GGK QVL**SP**DSL (conserved in human)
	KN motif and ankyrin repeat domain-containing protein 1 (MT)	KANK1	14	1352			
	KN motif and ankyrin repeat domain-containing protein 2 (MT)	KANK2	13	851		**R**SE**L**C**L**D**L** (NFAT4-like) [15,68]	
	catenin alpha-1/alpha-E-catenin	CTNNA1/CTNA1	1	906	detachment from P-catenin beta [67]		
	catenin alpha-2	CTNNA2/CTNA2	5	953			4 sites SAT**SP**IIL QAT**SP**TDE AVN**SP**VVS KHI**SP**VQA (conserved in human)
	catenin beta-1	CTNB1/CTNB1	3	781	YLD**S**GIH**S**GAT**T**TAP (S33 S37 T41) indirect [66]		1 site IMR**SP**QMV (conserved in human)
	fermitin family homolog 2	FERMT2/FERM2/ KIND2/MIG2	11	680			

helix along F-actin	tropomyosin alpha-1, beta, alpha-3, alpha-4 chains	TPM1, TPM2, TPM3, TPM4	0	284, 284, 285, 248		no site
troponin complex with tropomyosin-actin-myosin	troponin T slow skeletal, cardiac muscle (tropomyosin binding)	TNNT1, TNNT2	0	278, 298	released from ACT5-S232P (indirect JNK)? [43]	
	troponin T fast skeletal muscle (tropomyosin binding)	TNN3	1	269	released from ACT5-S232P (indirect JNK)? [43]	
	troponin I fast skeletal, cardiac muscle (inhibits myosin binding on actin)	TNNI2, TNNI3	0	182, 210		
	troponin I slow skeletal muscle (inhibits myosin binding on actin)	TNNI1	1 (KSP)	187		
	troponin C (Ca2+ binding)	TNNC1	1	161		
inhibition of actin/myosin binding	caldesmon	CALD1	6 (KSP)	793	KVT**<u>SP</u>**TKV (S789) [48]	
Ca2+ binding/alarmin	protein S100-A1 (MT, IF)	S100A1	0	94	activates JNK (RAGE) [40]	
	protein S100-A2, S100-A4, S100-A6, S100-A10, S100-A12	S100A2, A4, A6, A10, A12	0	92, 101, 100, 97, 92	activates JNK (RAGE) [40]	
	protein S100-A8 (MT, IF)	S100A8	0	93	activates JNK (RAGE) [40]	
	protein S100-A9 (MT, IF)	S100A9	1	114		
	protein S100-A11	S100A11	1	105	activates JNK (RAGE) [40]	
	protein S100-B (MT, IF)	S100B	0	92	activates JNK (RAGE) [40]	
	protein S100-P	S100P	0	95		
barbed-end directed motor	myosin-1, 2, 4, 13 (conventional II)	MYH1, MYH2, MYH13	1 (KTP)	1939, 1941, 1939, 1938		
	myosin-3, 8 (conventional II)	MYH3, MYH8	2 (KTP)	1940, 1937		
	myosin-9 (conventional II) ubiquitous (septin7, spectrin)	MYH9/NMIIa/M2A	2	1960		no site
	myosin-10 (conventional II) ubiquitous	MYH10/M2B	2	1976		no site
	myosin-6, 15 (conventional II)	MYH6, MYH15	0	1939, 1946		
	myosin-7, 7B (conventional II)	MYH7, MYH7B	3 (KSP, KTP)	1935, 1983		
	myosin-11 (conventional II)	MYH11	3	1972		
	myosin-14 (conventional II) ubiquitous	MYH14/M2C	9	1995		
	unconventional myosin-Ia	MYO1A	4 (KSP)	1043		
	myosin-IIIa	MYO3A	8	1616		
	unconventional myosin-Va	MYO5A	8	1855		2 sites ENI**<u>SP</u>**GQI AI**<u>SP</u>**TSA (conserved in human)

	unconventional myosin-VI (pointed-end directed) (DOCK7, septin)	MYO6	4 (KSP)	1294			
	unconventional myosin-VIIa	MYO7A	13	2215			
	unconventional myosin-IXa	MYO9A	18 (KSP)	2548			
	unconventional myosin-IXb	MYO9B	22 (KSP KTP)	2157		**RP**TS**L**A**L** (JIP-like) [15,68,69]	no site
	unconventional myosin-X	MYO10	14	2058			
	unconventional myosin-XV	MYO15	44 (KTP)	3530			
	unconventional myosin-XVI	MYO16	25	1858			
	unconventional myosin-XVIIIa	MYO18A/MY18A	7	2054			
	unconventional myosin-XIX	MYO19	10 (KSP)	970			
	myosin light chain 1/3 skeletal muscle isoform	MYL1/MLC1?	0	194			
	myosin regulatory light chain 2 ventricular/cardiac muscle isoform (septin/myosinII/ROCK=contraction)	MYL2/MLRV/MLC 2	0	166	LRD**T**FAA (T52) indirect (NET1/RhoA/ROCK/ MYPT) [49,50]		
	myosin light chain 3, 4	MYL3, MYL4	1	195, 197			
	myosin light chain 5, 6B (MYL6, spectrin)	MYL5, MYL6B	0	173, 208			
	myosin regulatory light chain 2 atrial isoform	MYL7/MLRA	2	175			
	myosin regulatory light polypeptide 9	MYL9	0	172			
	myosin regulatory light chain 12A, 12B	MYL12A, MYL12B MLC120/MRLC2?	0	171, 172			
	myosin regulatory light chain 2 skeletal muscle isoform	MLRS/MYLPF	0	169			
	mysosin light chain kinase	MYLK	16 (KTP)	1914			
	mysosin light chain kinase 2	MYLK2	10 (KTP)	596			
	mysosin light chain kinase 3	MYLK3	16 (KSP)	819			
	E3 ubiquitin-protein ligase MYLIP	MYLIP	4	445			
protein G	transforming protein RhoA	RHOA	2	193			
	cell division control protein 42 homolog	CDC42	3 (KTP)	191	activates JNK upon stress [34]		
	Ras-related C3 botulinum toxin substrate 1	RAC1	3	192			
GEF	TRIO and F-actin-binding protein	TARA/TRIOBP	66	2365			
	triple functional domain protein (GEF of RhoA, Rac)	TRIO	25 (KTP)	3097			2 sites GAV**SP**LNS (NDA**SP**PAS also in human)
	Rho guanine nucleotide exchange factor 18 (septin)	ARHGEF18/ SA-RhoGEF	22 (KSP)	1361			

	dedicator of cytokinesis protein 1/180 kDa protein downstream of CRK	DOCK1/DOCK180	13 (KTP)	1865	activates JNK (p130CAS, Rac1) [179,217]		
	dedicator of cytokinesis protein 2 (spectrin)	DOCK2	7 5KTP)	1830			
	dedicator of cytokinesis protein 3/ presenilin-binding protein (MT)	DOCK3/MOCA/ PBP	15 KSP)	2030	activates JNK (*Drosophila* Rac1) [177]		
	dedicator of cytokinesis protein 5 (MT, septin) (bone)	DOCK5	16 (KTP)	1870	activates JNK (p130CAS, Rac1) [176]	**RP**KS**L**Q**L** (JIP-like) [15,68]	
	dedicator of cytokinesis protein 7 (MYO6, septin)	DOCK7/Zir2	23	2140	activates JNK (Rac1) [178]	**RP**AS**L**N**L** (JIP-like) [68]	
	dedicator of cytokinesis protein 8 (Cdc42) (septin MT)	DOCK8/Zir3	13 (KSP)	2099			
	rhotekin	RTKN	9	563			
	isoform 2 of neuroepithelial cell-transforming gene 1	ARHG8/ARHGEF8 /NET1A	7	542	NLI**SP**VRN (S52 isoform2) nuclear to cytosolic localization [49,50]		
GAP	SLIT-ROBO Rho GTPase-activating protein 1	SRGP1/SRGAP1	10	1085			
	SLIT-ROBO Rho GTPase-activating protein 2	SRGP1/SRGAP2	14	1071			
	SLIT-ROBO Rho GTPase-activating protein 3	SRGP1/SRGAP3	12	1099			
scaffold	Ras GTPase-activating-like protein IQGAP1 (MT, FA, septin)	IQGAP1/IQGA1	8 (KTP)	1656			
	limatin/actin-binding LIM protein family member 1	LIMAB1/abLIM1/ ABLM1	12	778			2 sites IPG**SP**GHT YD**SP** (conserved in human)
	actin-binding LIM protein 2	abLIM2/ABLM2	10 (KSP)	611			3 sites QHP**SP**TSV (TSG**SP**SR TS**SP**SSA also in human)
	LIM domain and actin-binding protein 1	LIMA1/EPLIN	10 (KSP)	759			4 sites KPL**SP**SLR **SP**LEP (KPL**SP**DAR TPH**SP**GVE also in human)
	PDZ and LIM domain protein 7	PDLIM7/ENIGMA	7 (KTP)	457			1 site AL**TP**PAD (not conserved in human)
kinase/phosphatase	LIM domain kinase-1 (cofilin kinase)	LIMK1	9	647			
	LIM domain kinase-2 (cofilin kinase)	LIMK2	5 (KSP)	638			
	protein phosphatase slingshot homolog 1 (cofilin phosphatase)	SSH1	16 (KSP)	1049			no site

		neurabin-1	NEB1	12	1098			4 sites DSL**SP**R RP**SP**GEV (T**SP**DAS EAV**SP**TVS also in human)
		neurabin-2/spinophilin (PP1-mediated deP of doublecortin JNK site [95])	NEB2	5	817			no site
		myosin phosphatase targeting subunit 1/protein phosphatase 1 regulatory subunit 12A	MYPT1/PPP1R12A	12 (KSP)	1030			2 sites TPT**SP**VKK TKI**SP**K (but TPT**SP**IKK in human)
		myosin phosphatase targeting subunit 2/protein phosphatase 1 regulatory subunit 12B	MYPT2/PPP1R12B	14	982			
		myosin phosphatase targeting subunit 3/protein phosphatase 1 regulatory subunit 16A	MYPT3/PP16A/ PPP1R16A	9	528			
		mammalian STE20-like protein kinase 1/serine threonine-protein kinase 4	MST1/STK4	3	487	activates JNK if actin disruption [38]		
		mammalian STE20-like protein kinase 2/serine threonine-protein kinase 3	MST2/STK3	3	491			
		STE20-like serine/threonine-protein kinase/serine threonine-protein kinase 2	SLK/STK2	2	1235			
		nck-interacting kinase/mitogen-activated protein kinase kinase kinase kinase 4	NIK/MAPK4/M4K4	11 (KSP)	1239			
		NIK-related kinase/NIK-like embryo-specific kinase	NRK/NESK	13 (KTP)	1582			
		serine/threonine-protein kinase PAK 1/p21-activated kinase 1	PAK1	10	545			
		Rho-associated protein kinase 1	ROCK1	5	1354			
**MAP/ microtubule**	structural MAP	microtubule-associated protein 1B	MAP1B/neuraxin (heavy + LC1 chain)	79 (KSP KTP)	2468	AST**SP**SLS, STI**SP**PSS (S25 S1201 rat) [88]	JBD [69]	4 sites EDF**SP**EK (ETE**SP**SQE SA**TP**VDE TEL**SP**SFI also in human)
		microtubule-associated protein 1A	MAP1A (heavy + LC2 chain)	56 (KSP)	2803			31 sites (including 8 sites VLS**SP**EDL GFK**SP**PCE ATH**TP**FHQ SL**SP**EDA ET**SP**TRE LAE**SP**VGL PPA**SP**PEM QLP**SP**AEP conserved in human)
		microtubule-associated protein 1S	MAP1S/MAP8	29 (KSP KTP)	1059			
		microtubule-associated protein 2	MAP2	43 (KSP)	1827	SSR**TP**G**TP**G**TP**SYP (T1619 T1622 T1625 rat) [89]		1 site QAH**SP**SR (conserved in human)
		microtubule-associated protein 4 (septin)	MAP4	19	1152			10 sites PCV**SP**EVT SEG**SP**DTD DV**SPSP**ET NSM**TP**PSD

							DEG**SP**LEK DIA**TP**PNK RT**SP**SKP AT**SP**STL NT**TP**TGA (not conserved in human, but DIT**TP**PNK)
	microtubule-associated protein 6	MAP6/STOP	5	813			4 sites TTE**SP**SAT GQ**SP**TAP AQ**SP**LLP IEG**SP** (not conserved in human)
	ensconsin/epithelial microtubule-associated protein of 115 kDa	MAP7/EMAP115	14	749			
	microtubule-associated protein 9/ aster-associated protein	MAP9/ASAP	6 (KSP)	647			
	microtubule-associated protein 10/ microtubule regulator of 120 Kda	MAP10/MRT120	17 (KSP)	905			
	microtubule-associated protein Tau/ neurofibrillary tangle protein/ paired helical filament-Tau	Tau/MAPT	25 (KSP KTP)	758	GYSSPG**SP**G**TP**GSR, DMVD**SP**QLA (S519 S522 S739) [96]	colocalization with JIP3, JIP4 [202]	
	microtubule-associated protein Tau isoform F (septin)	Tau-isoF	17 (KSP KTP)	441	GYSSPG**SP**G**TP**GSR, DMVD**SP**QLA (S202 S205 S422) [96]		
	stabilizer of axonemal microtubules 1	Saxo1	1	474			
	stabilizer of axonemal microtubules 2	Saxo2	2	398			
doublecortin family	doublecortin (actin, neurabin II)	DCX	9 (KSP)	365	QLS**TP**KSKQ**SP**IS**TP** T**SP**GSL (T326 S332 T336 S339) [90,91]	**K**VD**L**Y**L**P**L** (NFAT4-like) [15]	5 sites GLP**SP**THS SK**SP**ADS KQ**SP**IS**TP**T**SP**GS (conserved in human)
	serine/threonine-protein kinase DCLK1 (actin, neurabin II)	DCLK1	16 (KSP)	740		JIPs/JNK binding [94]	7 sites RSK**SP**AST GK**SPSPSP**T**SP**GS DY**SP**SSSETVR**SP**NS (conserved in human)
	serine/threonine-protein kinase DCLK2 (actin, neurabin II)	DCLK2	13 (KSP)	766		JIPs/JNK binding [94]	
	doublecortin domain-containing protein 1 (actin)	DCDC1	14 (KSP)	1783		JIPs/JNK binding [94]	
	doublecortin domain-containing protein 2 (actin)	DCDC2	3	476		JIPs/JNK binding [94]	
	doublecortin domain-containing protein 2B (actin)	DCD2B	5	349		JIPs/JNK binding [94]	
	oxygen-regulated protein 1 (actin)	RP1	13 (KSP)	2156		JIPs/JNK binding [94]	
	retinitis pigmentosa 1-like 1 protein (actin)	RP1L1	47 (KTP)	2400		JIPs/JNK binding [94]	

Microtubule-Associated Tumor Suppressor Protein (MATSP)	microtubule-associated tumor suppressor 1	ATIP3/MTUS1	20 (KSP KTP)	1270			
	RAS Association domain Family 1A	RASSF1A	1	344			
	von Hippel–Lindau disease tumor suppressor	VHL	1	213			
	neurofibromin 2 (moesin-ezrin-radixin-like protein) (spectrin)	merlin/NF2	2	595			
	ubiquitin carboxyl-terminal hydrolase CYLD/cylindromatosis tumor suppressor	CYLD	8	956			
	adenomatous polyposis coli protein (+TIP) (FI, actin)	APC	56 (KSP KTP)	2843			18 sites (including 14 sites NIM**SP**GSS DNL**SP**K GRQ**SP**SQN TPK**SP**PEH SK**TP**PPP TIE**SP**PNE KPT**SP**VKP HGL**SP**DSE LG**SP**FHL**TP**DQ ASK**SP**SEG ATT**SP**R PNL**SP**TIE SGR**SP**TGN conserved in human)
	adenomatous polyposis coli protein 2 (actin)	APCL/APC2	42 (KTP)	2303		**RP**SR**L**D**L** (JIP-like) [15,68]	6 sites SR**TP**PAP R**SP**LAT GS**SP**EDS (PEG**SP**VHG IKL**SP**TYQ SST**SP**SLE conserved in human)
	breast cancer type 1 susceptibility protein/RING-type E3 ubiquitin transferase	BRCA1/RNF53	16 (KTP)	1863			
	leucine zipper putative tumor suppressor 1	LZTS1/FEZ1	4	596			
	fragile histidine triad/ bis(5'-adenosyl)-triphosphatase	Fhit	0	147			
+TIP	end-binding protein 1/ microtubule-associated protein RP/EB (septin, anchyrin-G)	EB1/MARE1	0	268			
	end-binding protein 2/ microtubule-associated protein RP/EB 2	EB2/MARE2	4	327			
	end-binding protein 3/ microtubule-associated protein RP/EB 3 (anchyrin-G)	EB3/MARE3	2	281			1 site QRT**SP**TGP (conserved in human)
	cytoplasmic linker protein 170, CAP-Gly domain-containing linker protein 1 (mDia actin, septin)	CLIP-170/ CLIP1/Restin	9 (KTP)	1438	ALK**TP**TAV, ASS**TP**SSE, RAT**SP**LCT (T25 T45 S147) [122]		2 sites AA**SP**LST (**SP**SAS also in human)

	cytoplasmic linker protein 115, CAP-Gly domain-containing linker protein 2	CLIP-115/CLIP2	7	1046	4 sites GTA**TP**PLT PST**SP**AK LTH**SP**SSS NRH**SP**GPE (conserved in human)
	CLIP-associating protein 1	CLASP1	16	1538	2 sites T**SP**LT**SP**TN (conserved in human)
	CLIP-associating protein 2	CLASP2	14	1294	1 site LHS**SP**R (but THS**SP**R in human)
	SLAIN motif-containing protein 2	SLAIN2	25	582	
	MCAK/kinesin like protein (depolymerizing kinesin) (septin)	MCAK/KIF2C	3	725	
	proline/serine-rich coiled-coil protein 1	DDA3/PSCR1	14 (KSP)	363	
	transforming acidic coiled-coil-containing protein 1	TACC1	18 (KSP)	805	
	transforming acidic coiled-coil-containing protein 2	TACC2	75 (KSP KTP)	2948	9 sites MQE**SP**TLS FN**SP**SEE GEE**SP**VPS (VQN**SP**PVG PA**SP**PR**SP**AE PS**SP**SK**SP**AS PVK**SP**PVR also in human)
	transforming acidic coiled-coil-containing protein 3 (spectrin)	TACC3	18	838	
	cytoskeleton-associated protein 5	CKAP5/TOG	9 (KSP)	2032	
	small kinetochore-associated protein/kinastrin	SKAP	0	316	
	GAS2-like protein 1 (actin)	GA2L1/Gas2L1/ GAR22	23	681	
	GAS2-like protein 2 (actin)	GA2L2/Gas2L2	19	880	
	GAS2-like protein 3 (actin)	GA2L3/Gas2L3	14 (KSP KTP)	694	
spectraplakin (spectrin family)	dystonin, bulbous pemphigoid antigen (actin, IF)	DST/BPAG1/BP230	45 (KSP KTP)	7570	
	microtubule-actin cross-linking factor 1/actin crosslinking family 7 (actin, IF)	MACF1/ACF7	36 (KSP KTP)	7388	
dynactin complex	dynactin subunit-1/p150-glued (SEPT9, spectrin)	DCTN1	13 (KSP)	1278	3 sites DTT**SP**ETP LT**SP**GA PLP**SP**SKE (conserved in human)
	dynactin subunit-2/p50-dynamitin (spectrin)	DCTN2	4	401	1 site G**TP**PDS (conserved in human)
	dynactin subunit-3/p22 (spectrin)	DCTN3	0	186	
	dynactin subunit-4/p62 (ankyrin-B)	DCTN4	1	460	
	dynactin subunit-5 /p25 (spectrin)	DCTN5	0	182	
	dynactin subunit-6 (spectrin)	DCTN6	2	190	
	actin related protein-1/centractin (spectrin)	ARP1/CTRN1/ACT R1A/ACTZ	0	376	

anchoring membrane	LL5alpha/pleckstrin homology-like domain family B member 1	LL5A/PHLDB1	35	1377			2 sites SP**SP**TLG SPL**SP**VAN (but SPL**SP**MAN in human)
	LL5beta/pleckstrin homology-like domain family B member 2 (actin)	LL5B/PHLDB2	19 (KSP KTP)	1253			
	liprin-alpha-1/protein tyrosine phosphatase receptor type f polypeptide-interacting protein alpha-1	PPFIA1/LIPA1	11	1202			
	liprin-alpha-3/protein tyrosine phosphatase receptor type f	PPFIA3/LIPA3/ PTPRF	14	1194			3 sites QAQ**SP**GGV GD**TP**PP**TP**R (conserved in human)
	disks large homolog 1/synapse-associated protein 97 (spectrin?)	DLG1/SAP97	11	904			2 sites ERI**SP**QVP SHI**SP**IK (but EHI**SP**QIT in human)
	disks large homolog 2/postsynaptic density protein 93/channel-associated protein of synapse-110	DLG2/PSD93/ chapsyn-110	7	870			5 sites SHI**SP**LK PI**SP**GRY**SP**IP KPA**SP**R HY**SP**VEC (conserved in human)
	disks large homolog 3/synapse-associated protein 102	DLG3/SAP102	6	817			1 site TRY**SP**IPR (conserved in human)
	disks large homolog 5/placenta and prostate DLG	DLG5/PDLG	20	1919			2 sites GSL**TP**PKP (AP**SP**PPL also in human)
-TIP	calmodulin-regulated spectrin-associated protein 1	CAMSAP1	21 (KSP KTP)	1602			
	calmodulin-regulated spectrin-associated protein 2	CAMSAP2	27 (KSP KTP)	1489	activates Rac1/JNK [78]		
	calmodulin-regulated spectrin-associated protein 3 (actin/ACF7)	CAMSAP3	35	1249			
	A-kinase anchor protein 6 (nesprin)	AKAP6/AKAP100	28	2319		**R**SK**L**C**L**V**L** (NFAT4-like) [15,68,69]	
	A-kinase anchor protein 9	AKAP9/AKAP350/ AKAP450	10	3907			
	pericentrin	PCNT	25 (KSP)	3336			
	CDK5 regulatory subunit-associated protein 2	CDK5RAP2/ CEP215	11 (KTP)	1893			
	mozart-1/mitotic-spindle organizing protein 1	MZT1	0	82			
	mozart-2/mitotic-spindle organizing protein 2A, 2B	MZT2A, MZT2B	1 (KSP), 1(KSP)	158, 158			
	neural precursor cell expressed, developmentally down-regulated 1	NEDD1	11 (KSP)	660			
	myomegalin/phosphodiesterase 4D-interacting protein	PDE4DIP/MMGL/ MYOME	25 (KTP)	2346			
	ninein/GSK3B-interacting protein	NIN	12	2090			

nucleation (centrosomal)	gamma-tubulin complex component 2/gamma-ring complex protein 103 kDa	TUBGCP2/ hGrip103	4	902			
	gamma-tubulin complex component 3/gamma-ring complex protein 104 kDa	TUBGCP3/ hGrip104	7 (KSP)	907			
	gamma-tubulin complex component 4/gamma-ring complex protein 76 kDa	TUBGCP4/hGrip76	3 (KTP)	667			
	gamma-tubulin complex component 5	TUBGCP5	5	1024			
	gamma-tubulin complex component 6	TUBGCP6	17	1819			
	pericentriolar material 1 protein	PCM1	19 (KTP)	2024			3 sites PLT**TP**LLD (NDI**SP**ESS G**SP**AGE also in human)
	centrosomal CEP170/KARP-1-binding protein (septin)	CEP170	23 (KTP)	1584			
	centrosome-associated protein 350	CAP350/CEP350	39 (KSP KTP)	3117			
nucleation (non centrosomal)	targeting protein for Xklp2	Tpx2	10 (KSP KTP)	747			
	HAUS augmin-like complex subunit 1	HAUS1	1	279			
	HAUS augmin-like complex subunit 2	HAUS2	0	235			
	HAUS augmin-like complex subunit 3	HAUS3	2 (KTP)	603			
	HAUS augmin-like complex subunit 4	HAUS4	1	363			
	HAUS augmin-like complex subunit 5	HAUS5	4	633			
	HAUS augmin-like complex subunit 6	HAUS6	17	955			
	HAUS augmin-like complex subunit 7	HAUS7	2	368			
	HAUS augmin-like complex subunit 8	HAUS8	5 (KSP KTP)	410			
severing	fidgetin	FIGN	13	759			
	katanin p60 ATPase-containing subunit A1	KTNA1/KATNA1	5 (KSP)	491			
	katanin p80 WD40 repeat-containing subunit B1	KTNB1/KATNB1	8	655			
	katanin p60 ATPase-containing subunit A-like 1	KATL1/KATNAL1	1	490			
	katanin p60 ATPase-containing subunit A-like 2	KATL2/KATNAL2	1	538			
	KATNB1-like protein 1	KTBL1/KATNBL1	4 (KSP)	304			
	spastin (ESCRT)	SPAST/SPG4	4	616			
dimer binding	stathmin-1	STMN1	2	149	LIL**SP**RSK, FPL**SP**PKK (S25 S38) [105]	KKKD**L**S**L** [105]	1 site FPL**SP**PKK (conserved in human)
	stathmin-2, superior cervical ganglion-10 protein	STMN2/SCG10	2	179	KPP**SP**ISE TLA**SP**KKK (S62 S73) [105]	KKKD**L**S**L** [105]	1 site KPP**SP**ISE (conserved in human)

	stathmin-3	STMN3/SCLIP	3 (KSP)	180	ILK**SP**SDL (S60) [105]	KKKDTS**L** [105]	4 sites ILK**SP**SDL**SP**E**SP**VLS**SP**PK (but ILK**SP**SDL**SP**E**SP**MLS**SP**PK in human)
	stathmin-4	STMN4/RB3	0	189			no site
	gephyrin /molybdopterin molybdenumtransferase	GPHN/GEPH	10	736			
kinesin motor	kinesin heavy chain isoform 5A (spectrin)	KIF5A	6	1032			
	kinesin-1 heavy chain (spectrin ankyrin, IF)	KIF5B/uKHC/ KINH	3	963	PAA**SP**THP (S934 mouse brain)? [128]. Activates JNK [76,77]		1 site PAA**SP**THP (conserved in human)
	kinesin heavy chain isoform 5C	KIF5C	4	957	FVS**SP**EEV (S176 mouse brain) (PAA**SP**TAV (S934)?) [110, 128]		
	kinesin-like protein KIF1A, (spectrin)	KIF1A	22 (KTP)	1690	P-SYT4 = detach from KIF1A [111]		2 sites RPA**SP**EPE DAG**SP**GMQ (conserved in human)
	kinesin-like protein KIF1B (spectrin)	KIF1B	28 (KSP KTP)	1817			
	kinesin-like protein KIF1C	KIF1C	13 (KTP)	1103			
	kinesin-like protein KIF2A, kinesin-like protein KIF2B (depol) (spectrin)	KIF2A, KIF2B	7, 7 (KSP)	706, 673			
	kinesin-like protein KIF3A, kinesin-like protein KIF3B (spectrin)	KIF3A, KIF3B	2, 1	699, 747	colocalization MLK2/P-JNK/kif3a [203]		
	chromosome-associated kinesin KIF4A	KIF4A	8 (KTP)	1232			
	kinesin-like protein KIF6	KIF6	6	814			
	kinesin-like protein KIF7	KIF7	5	1343			
	kinesin-like protein KIF9	KIF9	4	790			
	kinesin-like protein KIF11	KIF11/Eg5	5	1056			
	kinesin-like protein KIF13A	KIF13A	13	1805			
	kinesin-like protein KIF13B/GAKIN	KIF13B/GAKIN	18 (KSP)	1826			
	kinesin-like protein KIF14 (FA)	KIF14	8 (KSP)	1648			
	kinesin-like protein KIF15	KIF15	6 (KTP)	1388	activates JNK [79]		
	kinesin-like protein KIF16B	KIF16B	5 (KSP)	1317			
	kinesin-like protein KIF17 (SEPT9)	KIF17	3	1029			
	kinesin-like protein KIF18A, kinesin-like protein KIF18B (depol)	KIF18A, KIF18B	3 (KSP), 15	898, 252			
	kinesin-like protein KIF19 (depol)	KIF19	7	998			
	kinesin-like protein KIF20A	KIF20A	7	890			
	kinesin-like protein KIF21A, kinesin-like protein KIF21B	KIF21A, KIF21B	12, 16 (KSP)	1674, 1637			

	kinesin-like protein KIF22	KIF22	6	665		
	kinesin-like protein KIF23	KIF23	8 (KTP)	960		
	kinesin-like protein KIF24	KIF24	25	1368		
	kinesin-like protein KIF25 (minus end)	KIF25	2	384		
	kinesin-like protein KIF28P	KIF28P	4	967		
	kinesin light chain 1 (spectrin)	KLC1	2	573	JIP binding [118]	1 site VD**SP**TVT (conserved in human)
	kinesin light chain 2 (spectrin)	KLC2	5	622		1 site EDA**SP**NEE (conserved in human)
	kinesin light chain 3 (spectrin)	KLC3	3	504		
	kinesin light chain 4 (spectrin)	KLC4	2	619		
	centromere-associated protein E (kinesin) (spectrin, septin)	CENPE	7 (KSP)	2701		
dynein motor	cytoplasmic dynein 1 heavy chain 1 (spectrin)	DYHC1/DYNC1H1	23 (KSP KTP)	4646		
	cytoplasmic dynein 2 heavy chain 1	DYHC2/DYNC2H1	21	4306		
	cytoplasmic dynein 1 intermediate chain 1 (SEPT9 spectrin)	DC1I1/DYNC1I1	10	637		4 sites IGI**SP**EPP VP**TP**M**SP**SS ETQ**TP**LAT (conserved in human)
	cytoplasmic dynein 1 intermediate chain 2 (spectrin)	DC1I2/DYNC1I2	9	638		1 site PPM**SP**SSK (conserved in human)
	cytoplasmic dynein 2 intermediate chain 1	DC2I1/DYNC2I1	9	1066		
	cytoplasmic dynein 2 intermediate chain 2	DC2I2/DYNC2I2	4	536		
	cytoplasmic dynein 1 light intermediate chain 1 (spectrin)	DC1L1/DYNC1LI1/ LIC1	11	523		5 sites GS**SP**PGL FPA**SP**QRR V**SP**T**TP**P**SP**TE (but V**SP**T**TPTSP**TE in human)
	cytoplasmic dynein 1 light intermediate chain 2 (spectrin)	DC1L2/DYNC1LI2	8	492		1 site CQG**SP**QR (conserved in human)
	cytoplasmic dynein 2 light intermediate chain 1	DC2L1/DYNC2LI1	3	351		
	dynein light chain 1 cytoplasmic, dynein light chain 2 cytoplasmic (spectrin)	DYNNL1/DLC1, DYNNL2/DLC2	0	89, 89		no site
	dynein heavy chain 12 (axonemal)	DYH12/DNAH12	19	3092	**K**EA**L**N**L**K**L** (NFAT4-like) [15,68]	
dynein regulation	centromere-associated protein F	CENPF	22 (KTP)	3114		
	nuclear distribution protein nudE-like 1 (ankyrin-G)	NDEL1/NUDEL/ NUDE2	5	345		3 sites APS**SP**TLD FP**SP**K FGT**SP**LTP (conserved in human)

	platelet-activating factor acetylhydrolase IB subunit beta/lissencephaly-1 protein (actin)	PAFAH1B1/LIS1	1	410			
	serine-rich coiled-coil domain-containing protein 1	CCSER1/FAM190A	15	900		**K**QN**L**S**L**K**L** (NFAT4-like) [15,68]	
transported cargo	amyloid-beta precursor protein	APP (isoform 695)	3	695	AAV**TP**EER (T668) EFV kin1? [112]		
	amyloid-like protein 1	APLP1	7	650			
	amyloid-like protein 2	APLP2	4	425	PML**TP**EER (T736) [115]		
	synaptotagmin-4	SYT4	5 (KTP)	425	ESV**SP**ESK (S135) = released from kif1A [111]		
	Bcl-2-like protein11 isoform L	BimL/B2L11	3	138	STQ**TP**SPP (T56) = released from dynein [116]		
	Bcl-2-modifying factor	Bmf	2	184	QTL**SP**ASP (S74) = released from DLC/dynein/ myosinV [116]		
	apoptosis regulator Bcl-2	BCL2	6	239	ART**SP**LQT PAL**SP**VPP (S70 S87) KHC/JNK dependent [76]		
	dynamin-1-like protein/dynamin-related protein 1 (mitochondria)	DRP1/DNM1L	4	736	MPA**SP**KQG KHC/JNK dependent (S616) [77]		
	CAP-Gly domain-containing linker protein 3	CLIP3/CLIPR59	7 (KTP)	547			3 sites EAP**SP**TQE KK**SP**S**SP**SL (conserved in human)
	CAP-Gly domain-containing linker protein 3	CLIP4/RSNL2	5 (KSP)	705			
	ubiquitin carboxyl-terminal hydrolase CYLD	CYLD	8	956			
post-transtional-modifying enzyme	alpha-tubulin N-acetyltransferase 1	ATAT/MEC17	7	421			
	histone deacetylase 6 (scaffolded by SEPT7)	HDAC6	13	1215	transcriptional repression by Rac/JNK [78]		1 site NPQ**SP**LQD (but NPQ**SP**PQD in human)
	tubulin-tyrosine ligase	TTL	2 (KTP)	377			

	tubulinyl-Tyr carboxypeptidase 1/ vasohibin-1	VASH1/TTCP1	5	365	
	tubulinyl-Tyr carboxypeptidase 2/ vasohibin-2	VASH2/VASHL	4 (KSP)	355	
	small vasohibin-binding protein	SVBP	0	66	
	tubulin polyglutamylase 1 (septin)	TTLL1	5 (KSP)	423	
	tubulin polyglutamylase 2	TTLL2	6	592	
	tubulin monoglycylase 3	TTLL3	7	772	
	tubulin polyglutamylase 4	TTLL4	18 (KSP)	1199	
	tubulin polyglutamylase 5	TTLL5	9	1281	
	tubulin polyglutamylase 6	TTLL6	9 (KSP)	843	
	tubulin polyglutamylase 7	TTLL7	7 (KSP KTP)	887	
	protein monoglycylase 8	TTLL8	7	850	
	protein polyglutamylase 9	TTLL9	2	439	
	protein polyglycylase 10	TTLL10	7 (KSP KTP)	673	
	protein polyglutamylase 11, tubulin-tyrosine ligase-like 12 (septin)	TTLL11, TTLL12	5	800, 644	
	protein polyglutamylase 13	TTLL13	4	815	
	cytosolic carboxypeptidase 1 (septin)	CCP1/CBPC1/ AGTPBP1	5	1226	
	cytosolic carboxypeptidase 2	CCP2/CBPC2/ AGBL2	5	902	
	cytosolic carboxypeptidase 3	CCP3/CBPC3/ AGBL3	8	1001	
	cytosolic carboxypeptidase 4	CCP4/CBPC4/ AGBL1	6	1112	
	cytosolic carboxypeptidase-like protein 5	CCP5/CBPC5/ AGBL5	16 (KSP)	886	
	serine/threonine-protein kinase MARK1	MARK1	7	795	
	serine/threonine-protein kinase MARK2	MARK2	11	788	
	MAP/microtubule affinity-regulating kinase 3	MARK3	7	753	1 site TGQ**SP**HHK (conserved in human)
	MAP/microtubule affinity-regulating kinase 4	MARK4/MARKL1	8	752	1 site R**SP**TST (conserved in human)
	microtubule-associated serine/threonine-protein kinase 1 (actin syntrophin?)	MAST1	49 (KTP)	1570	
	microtubule-associated serine/threonine-protein kinase 2 (actin syntrophin?)	MAST2	50 (KTP)	1798	
	N-terminal acetyl transferase	Nat9	0	207	
tubulin chaperoning	tubulin-specific chaperone A	TBCA	0	108	

		tubulin-specific chaperone B/cytoskeleton-associated protein 1, tubulin-specific chaperone C	TBCB/CKAP1, TBCC	2, 2	244, 346		1 site YEI**SP**EAY (not conserved in human)
		tubulin-specific chaperone D	TBCD	4	1192		
		tubulin-specific chaperone E	TBCE	1	527		
	GEF	Rho guanine nucleotide exchange factor 2 (sequestered by MT)	ARHG2/ARHGEF2 /GEF-H1	9	986	if released from MT = activates RhoA/ MKK4/JNK [84]	
	stabilization golgian MT	golgi reassembly-stacking protein 1	GRASP65/GORS1	10	440	GPG**SP**SHS (S274) [123]	
	spindle	protein regulator of cytokinesis 1	PRC1	5 (KTP)	620		
		protein zwilch homolog (septin)	ZWILCH	2	591		
		kinetochore protein NDC80 homolog	NDC80/HEC	2	642		
		spindle and kinetochore-associated protein 1	SKA1	0	255		
		spindle and kinetochore-associated protein 2	SKA2	1	121		
		spindle and kinetochore-associated protein 3	SKA3	14 (KSP KTP)	412		
		kinetochore protein Nuf2	NUF2	1	464		
		kinetochore protein Spc24	SPC24	0	197		
	nucleation/bundling	tubulin polymerization-promoting protein	TPPP/p25/TPPP1	4 (KSP)	219		
		tubulin polymerization-promoting protein family member 2	TPPP2	1 (KSP)	170		
		tubulin polymerization-promoting protein family member 3 (catenin)	TPPP3	0	176		
		tripartite motif-containing protein 46 (ankyrin-G)	TRIM46/TRI46	8	759		
**IFAP/ intermediate filament**	plakin (spectrin family)	plectin (MT, actin)	PLEC	12 (KTP)	4684		
		desmoplakin (desmosome) (MT)	DSP	5	2871		
		envoplakin (desmosome)	EVPL	13 (KSP)	2032		
		periplakin (desmosome)	PPL	6	1756		
		epiplakin	EPPK	18	5088		
	nuclear linker (spectrin family)	nesprin-1 (actin, MT/KLC?)	SYNE-1	32 (KTP)	8797		
		nesprin-2 (actin, MT/KLC?)	SYNE-2	30 (KSP)	6885		
		nesprin-3 (plectin/IF, MACF1-BPAG1/MT)	SYNE-3	3	975		

	nesprin-4 (MT/KIF5B)	SYNE-4	4	404		
nuclear linker	emerin (lamin) (nuclear actin, spectrin)	EMD/STA	1	254		
	barrier-to-autointegration factor	BAF/BANF1	0	89		
	zinc finger protein 239	MOK2/ZNF239/ HOK2	4	458	EAS**SP**ISR ELA**SP**LLN (Ser38 Ser129) = released from lamin A/C [153]	
muscle	myospryn/cardiomyopathy-associated protein 5 /dystrobrevin-binding protein 2	CMYA5/SPRYD2/ DTNBP2	67 (KSP)	4069		
	myotubularin	MTM1	3	603		
	titin/connectin	TTN	378 (KSP KTP)	34,350		
	nebulin	NEBU/NEB	90 (KTP)	6669		
	tripartite motif-containing protein 55/muscle-specific RING finger protein 2 (MT, actin, septin)	TRIM55/MURF2/ TRI55	3	548		
keratin crosslinker	trichohyalin	TCHH/TRHY/THL	2	1943		
	filaggrin (keratin condensation, terminal differentation)	FILA/FLG	11 (KSP)	4061		
deiminase	peptidylarginine deiminase 1/protein-arginine deiminase type-1 (keratin)	PAD1/PADI1	5	663		
	peptidylarginine deiminase 2/protein-arginine deiminase type-2 (vimentin, NEF) (myosin, tubulin)	PAD2/PADI2	6	665		
	peptidylarginine deiminase 3/protein-arginine deiminase type-3 (HF, keratin)	PAD3/PADI3	9	590		
	peptidylarginine deiminase 4/protein-arginine deiminase type-4 (IF, vimentin)	PAD4/PAID4	8 (KTP)	663		
	peptidylarginine deiminase 6/protein-arginine deiminase type-6 (vimentin) (tubulin)	PAD6/PADI6	7	694		
gamma-glytamyltransferase	protein-glutamine gamma-glutamyltransferase 2/transglutaminase 2	TGM2/TGase-2/ TG2	4	687	activates DLK [63,64,145]	
heat shock protein	αB-crystallin/beta-crystallin A3 (keratin)	CRBA1/CRYBA1/ HSPB5	1	215		
	heat shock protein 27	HSP27	2	205	RAL**S**RQL (S78) indirect [152]	
GEF	Rho guanine nucleotide exchange factor 40 (keratin 8/18, hemidesmosome)	Solo/ARHGEF40/ ARH40	30	1519		
Cdc42 regulator	breast carcinoma-amplified sequence 3 (vimentin) (MT)	BCAS3/(rudhira)/ GAOB1?	17 (KTP)	928		1 site MAE**SP**SR (conserved in human)

**SeptAP/septin**	LRCH3/DOKC7/MYO6 complex	DISP complex protein LRCH3 (DOCK7, myosin MYO6)	LCRH3	7	777			
		Four and a half LIM domains protein (actin tension)	FHL2	1	279			
	planar polarity/ciliogenesis	WD repeat-containing and planar cell polarity effector protein fritz homolog (actin)	WDPCP	3	746			
	exocyst, vesicle tethering	exocyst complex component 3	EXOC3/SEC6	4	745			
		exocyst complex component 4	EXOC4/SEC8	3	974	binding to JIP4/JLP = inhibits MKK4/JNK [163]		1 site DA**SP**GPL (not conserved in human)
		exocyst complex component 7	EXOC7/EXO70	7	735			
	SNARE, vesicle fusion	syntaxin-1A (tSNARE)	STX1A	1	288		RIQRQ**L**E**I** [165]	
		syntaxin-2 (tSNARE)	STX2	0	288		RIQRQ**L**E**I** [165]	
		vesicle-trafficking protein SEC22b/ER-Golgi SNARE of 24 kDa	SEC22B/ERS24/ SC22B	1	215			
		synaptosomal-associated protein 23 (tSNARE) (myosin MYH9)	SNAP23/SNP23	1	211			
		synaptosomal-associated protein 25 (tSNARE)	SNAP25/SNP25	0	206		RRM**L**Q**L** [165]	no site
		syntaxin-binding protein 1/UNC18a	Munc18-1/ STXBP1/N-SEC1	5	594			no site
		vesicle-associated membrane protein 1/synaptobrevin-1 (vSNARE)	VAMP1	0	118			
		vesicle-associated membrane protein 2/synaptobrevin-2 (vSNARE) (myosin MYH9)	VAMP2	0	116			
		vesicle-fusing ATPase/N-ethylmaleimide-sensitive fusion protein	NSF	4	744			
		synaptophysin/major synaptic vesicle protein p38	SYP/SYPH	0	313			
	chaperone	alpha-synuclein (MT)	SNCA/SYUA/ PARK1/NACP	0	140	colocalization P-◎SYN, P-Tau, P-JNK mitochondrial [104]		
		beta-synuclein	SNCB/SYUB	0	134			
	vesicle trafficking fusion recycling	synaptojanin-1 (phosphatase)	SYNJ1	23 (KSP)	1573			3 sites T**SP**CQ**SP**T GRL**TP**ESQ (conserved in human)
		synapsin-2 (actin)	SYN2	5 (KTP)	582			2 sites **TP**AL**SP**QR (conserved in human)

	vesicle-associated membrane protein (VAMP)-associated protein B/C	VAPB/VAMP-B/ VAMP-C	2	243			1 site PLT**SP**LDD (not conserved in human)
	synaptogyrin-3	SYNGR3/SNG3	2	229			
	synaptotagmin-1 (Ca2+)	SYT1/p65/SVP65	1	422			
	Munc18-1-interacting protein 1/amyloid-beta A4 precursor protein-binding family A	Mint1/APBA1	11 (KTP)	837			7 sites P**TP**GGGHPD**SP**GL VVG**TP**GGS (ESD**SP**EKE IR**SP**Y**TP**DE GSS**SP**LGA also in human but P**TP**AGGRPD**SP**GL)
	neuronal Munc18-1-interacting protein 2/amyloid-beta A4 precursor protein-binding family A member 2	Mint2/APBA2	8	749		**RP**KS**L**N**L** (JIP-like) [68]	no site
	ADP-ribosylation factor 1 (actin)	ARF1	0	181			
	sortin-nexin 6	SNX6	0	406			
	dynamin 1 isoform 1, 3 (MT)	DYN1/DNM1 iso1, iso3	12, 10	864, 851			
adaptor protein complex coat	AP-2 complex subunit alpha-2/alpha2-adaptin/alpha-adaptin C	AP2A2	5	939			
	AP-3 complex subunit beta-2/beta-3B-adaptin	AP3B2	8 (KTP)	1082			
	AP-2 complex subunit gamma/clathrin coat assembly protein AP17/sigma2-adaptin	AP2S1/CLAPS2	0	142			
clathrin	clathrin heavy chain	CLTC/CLH17	6	1675			no site
post-transtional-modifying enzyme	E3 ubiquitin-protein ligase parkin/Parkinson disease protein 2	PRKN/PARK2	4	465			
	SH3 domain-containing kinase-binding protein 1/Cbl-interacting protein of 85 kDa	SH3K1/SH3KBP1/ CD2BP3/HSB1/ CIN85	7	665			
BORG	Cdc42 effector protein 2	Cdc42EP2/CEP2/ BORG1	6	210			
	Cdc42 effector protein 3 (actin, MT)	Cdc42EP3/CEP3/ BORG2	7 (KTP)	254			
	Cdc42 effector protein 5 (actin, MT)	Cdc42EP5/CEP5/ BORG3	2	148	inhibits JNK [170]		
	Cdc42 effector protein 4	Cdc42EP4/CEP4/ BORG4	4	356			
	Cdc42 effector protein 1	Cdc42EP1/CEP1/ BORG5	11	391			
nuclear	histone deacetylase complex subunit SAP18/sin3-associated polypeptide p18	SAP18/GIG38	1	153			
	serine/arginine-rich splicing factor 7	SRSF7	7	238			

		(MT Tau)					
		scaffold attachment factor B1	SAFB/SAFB1	3	915		1 site AAP**SP**EPR (but EAP**SP**EAR in human)
		protein BUD31 homolog	BUD31/EDG2	0	144		
		transformer-2 protein homolog alpha	TRA2A	10 (KSP)	282		
		protein Red/cytokine IK (MT kinetochore)	IK/RED	2 (KTP)	557		
		cell division cycle 5-like protein	CDC5L	12	802		
		paired amphipathic helix protein Sin3a	SIN3A	16	1273		
**ESCRT-III-associated**	ATPase	vacuolar protein sorting-associated protein 4A	VPS4A	4	437	if depleted: JNK activation [195]	
		vacuolar protein sorting-associated protein 4B	VPS4B	5	444		
	VPS4 cofactor	vacuolar protein sorting-associated protein VTA1	VTA1/LIP5	5 (KTP)	307		
	ESCRT recruitment	programmed cell death 6-interacting protein (cytokinesis) (MT)	PDCD6IP/ALIX	8	868	POSH binding: activates JNK *(Drosophila)* [194]	
	ESCRT-I	tumor susceptibility gene 101 protein, vps23 (MT, septin)	TSG101	1	390	recruited by p130cas/Crk [221]	
	ESCRT-II	vacuolar protein-sorting-associated protein 25, Vps25	EAP2/VPS25	1	176		
	IST1 (glycosylation)	alpha-1,3/1,6-mannosyltransferase ALG2	ALG2	2	416	POSH binding: activates JNK *(Drosophila)* [194]	
	CHMP1B binding	ubiquitin carboxyl-terminal hydrolase 8 (MT)	USP8/UBPY	13	1118		
	ESCRT-0/ESCRT-I	flotillin-1 (MT centrosome)	FLOT1	1	427		no site
		flotillin-2 (actin)	FLOT2	0	428		
**spectrin-associated**	membrane/spectrin/actin	ankyrin-1/R (erythrocyte) (MT, IF)	ANK1/ANKR	40	1881		
		ankyrin-2/B (MT)	ANK2/ANKB	87 (KSP KTP)	3957		
		ankyrin-3/G (neuron) (MT kinesin)	ANK3/ANKG	94 (KSP KTP)	4377		
		alpha-adducin (actin)	ADDA/ADD1	17 (KSP)	737		9 sites R**SP**G**TP**AGEGSG**SP**P EK**SP**PDQSAVPN**TP**PS**TP**VK (also VVT**SP**PPT GTC**SP**LR NGS**SP**K in human but R**SP**G**SP**VGEGTG**SP**P EK**SP**PDQPAVPHPPPS**TP**IK)
		beta-adducin	ADDB/ADD2	16 (KSP)	726		9 sites PG**SP**VKSTPA**SP**VQ**SP**TRAGTK **SP**AV**SP**SK PL**SP**EGSPSK**SP**SK

								(also SR**SP**STE conserved in human but PG**SP**AKSAPA**SP**VQ**SP**AKEAET K**SP**LV**SP**SK PM**SP**EGSPSK**SP**SK) (iso2 GPL**TP** not conserved in human)
		gamma-adducin (actin)	ADDG/ADD3	16 (KSP)	726			5 sites VL**SP**DGSPSK**SP**SK (also VIT**TP**PPP ILQ**SP**AFR VPL**SP**LK in human but VL**SP**EGSPSK**SP**SK)
		protein 4.1R/band4.1/erythrocyte membrane protein band 4.1 (actin, MT)	EPB41/E41P/4.1R	5	864			2 sites NGD**TP**THE DR**SP**RPT (conserved in human)
		protein 4.2/erythrocyte membrane protein band 4.2	EPB42/E42P	5	691			
		protein 4.1N/band 4.1-like protein-1/erythrocyte membrane protein band 4.1-like 1	EPB41L1/E41L1/ 4.1N	18 (KSP)	881			17 sites QEE**TP**QQP FMT**TP**PCI IED**SP**DRG (also VT**TP**V**TP**AG ERT**TP**SK LV**SP**EPP EVR**TP**TKI QET**TP**R S**SP**A**SPSP**KG**TP**E D**SP**DRGACS**TP** SL**SP**IIG TDP**SP**EER in human but IAT**TP**SIT)
		protein 4.1G/band 4.1-like protein-2/erythrocyte membrane protein band 4.1-like 2 (actin, MT)	EPB41L2/E41L2/ 4.1G	9	1005			
		protein 4.1B/band 4.1-like protein-3/erythrocyte membrane protein band 4.1-like 3 (actin)	EPB41L3/DAL/4.1B	12 (KSP)	1086			no site
		band 4.1-like protein-4A/erythrocyte membrane protein band 4.1-like 4A	EPB41L4A/NBL4	7 (KSP)	686			2 sites IAP**SP**VK SR**SP**DIQ (conserved in human)
		band 4.1-like protein-4B/erythrocyte membrane protein band 4.1-like 4B	EPB41L4B	21 (KSP)	900			
	gap junction	connexin-43	CXNK2	4	382	unbinding αII-spectrin-SH3i/ connexin-43 if JNK activated [189]		no site
**JNK pathway**	scaffold	JNK-interacting protein 1/Islet-brain 1 (MT kinesin)	JIP1/IB1	11	711	TGD**TP**GAE GEQ**TP**PHE SVS**SP**YES (T103 T205 S421) [117, 119]	RPK**RP**TT**L**N**L**F (JIP) [15]	2 sites GAA**SP**PAA HIA**SP**PNF (conserved in human)
		JNK-interacting protein 2/Islet-brain 2 (MT kinesin)	JIP2/IB2	16	824		HKH**RP**TT**L**R**L**T (JIP)	1 site LIP**SP**SIE (but LIP**SP**SVE in human)

	JNK-interacting protein 3/JNK-stress activated protein kinase -associated protein-1 (MT kinesin dynein, MYO5A)	JIP3/JSAP1	14 (KSP)	1335	AAA**TP**STTGTKSN**T** **P**TSSVPSAAV**TP**LN E (T266 T276 T287) [120]	RKE**RP**TS**L**N**V**F (JIP) [15,68]	5 sites TGS**SP**TQG SSS**SP**PPA HYK**SP**TTA QL**SP**NGG NKT**SP**TSG (conserved in human)
	JNK-interacting protein 4/JNK-associated leucine-zipper protein (MT kinesin dynein)	JIP4/JLP/SPAG9	14	1321			no site
	WD repeat-containing protein 62 (MT)	WDR62	28 (KSP KTP)	1518	LPQ**TP**EQE (T1053) [124] Activates JNK [127] On MT spindle [126]	**R**AN**L**R**L**T**L** (NFAT4-like) [15]	
	beta-arrestin-2/non-visual arrestin 3 (MT)	ARRB2	3	409	JNK3 scaffold [14,17,209]. On MTs [211]	LMSDRRS**L**H**L**E	
	filamins (cf actin ABP)	(cf actin ABP)	(cf actin ABP)	(cf actin ABP)			
adaptor	elongator complex protein 1 (tubulin)	ELP1/IKAP	9	1332			
	CAS scaffolding protein/breast cancer anti-estrogen resistance protein 1 (focal adhesion, actin, IF, ESCRT)	p130CAS/BCAR1	16	870	activates Rac1/JNK [217]		
	adapter molecule crk (p130CAS binding)	CRK	4	304	activates Rac1/JNK [217]		
	E3 ubiquitin-protein ligase SH3RF1/plenty of SH3s (ESCRT Alix, actin)	SH3RF1/SH3R1/ POSH1	16	888			
	receptor of activated protein C kinase 1 (MT, spectrin, IF, plectin)	RACK1	4	317	activates MAPKKKS/MKK7/ JNK [222]. Binds dynactin, β–actin [223,224]		
	GRIP1-associated protein 1	GRASP1/GRIPAP1	2	841			1 site LSS**SP**QAQ (conserved in human)
dual phosphatase	dual specificity protein phosphatase 1/MAP kinase phosphatase 1 (nucleus/perinucleus, cytoplasm) (JNK)	DUSP1/MKP1	6	367			
	dual specificity protein phosphatase 2 (nucleus/perinucleus) JNK	DUSP2/PAC1	2	314			
	dual specificity protein phosphatase 3/vaccinia H1-related phosphatase (nucleus, cytoplasm) JNK	DUSP3/VHR	2	185			
	dual specificity protein phosphatase 4/MAP kinase phosphatase 2	DUSP4/MKP2/VH2	8 (KTP)	394			

	(nucleus) (JNK)					
	dual specificity protein phosphatase 6/MAP kinase phosphatase 3 (nucleus, cytoplasm, filaments?) JNK	DUSP6/MKP3/ PYST1	6	381		
	dual specificity protein phosphatase 7 (cytoplasm) JNK	DUSP7/PYST2	7	419		
	dual specificity protein phosphatase 8 (cytoplasm MT) (JNK)	DUSP8/VH5/M3/6	16	625	partial localization with MTs [226]	
	dual specificity protein phosphatase 10/MAP kinase phosphatase 5 (nucleus, cytoplasm) (JNK)	DUSP10/MKP5	9	482		**RP**QD**L**N**L** (JIP-like) [15,68]
	dual specificity protein phosphatase 12 (nucleus) JNK	DUSP12/YVH1	2	340		
	dual specificity protein phosphatase 13A (scaffold)	DUSP13A/MDSP	2	188		
	dual specificity protein phosphatase 13B JNK	DUSP13B/SKRP4/ TMDP	1	198		
	dual specificity protein phosphatase 14/MAP kinase phosphatase 6 (nucleus, cytoplasm) JNK & TAK1	DUSP14/MKP6/ MKPL	1	198		
	dual specificity protein phosphatase 16/MAP kinase phosphatase 7 (cytoplasm) (JNK)	DUSP16/MKP7	10	665		
	dual specificity protein phosphatase 18 (nucleus, centrosome?) JNK	DUSP18/ LMWDSP20	2	188		
	dual specificity protein phosphatase 19 (scaffold, nucleus, cytoplasm)	DUSP19/DUSP17/ LMWDSP3/SKRP1	0	217		
	dual specificity protein phosphatase 22/JNK-stimulatory phosphatase-1 (scaffold, nucleus, cytoplasm)	DUSP22/JSP1/ LMWDSP2/MKPX	2	184		
	dual specificity protein phosphatase 23 (scaffold, nucleus, cytoplasm)	DUSP23/LDP3/ VHZ	0	150		
MAPKK	dual specificity mitogen-activated protein kinase kinase 4	MKK4/MAP2K4	7	399		**R**KA**L**K**L**N**F** (NFAT4-like) [15]
	dual specificity mitogen-activated protein kinase kinase 7	MKK7/MAP2K7	6	419		**R**ID**L**N**L**D**I** (NFAT4-like) [15]
MAPKKK	mitogen-activated protein kinase kinase kinase 1	MEKK1/MAP3K1	33	1512		**K**NS**M**T**L**D**L** (NFAT4-like) [15,68]
	mitogen-activated protein kinase kinase kinase 2 (FA)	MEKK2/MAP3K2	12	619		

		mitogen-activated protein kinase kinase kinase 3	MEKK3/MAP3K3	7 (KSP)	626		
		mitogen-activated protein kinase kinase kinase 4	MEKK4/MAP3K4	14 (KSP)	1608		
		mitogen-activated protein kinase kinase kinase 5/apoptosis signal-regulating kinase 1	MEKK5/MAP3K5/ ASK1	4 (KTP)	1374		
		mitogen-activated protein kinase kinase kinase 6/apoptosis signal-regulating kinase 2	MEKK6/MAP3K6/ ASK2	16	1291		
		mitogen-activated protein kinase kinase kinase 7/TGF-beta-activated kinase 1	MAP3K7/TAK1	2	606		
		mitogen-activated protein kinase kinase kinase 9/mixed lineage kinase 1	MAP3K9/MLK1	28 (KTP)	1104		2 sites S**SP**PAS TP**SP**SRD (conserved in human)
		mitogen-activated protein kinase kinase kinase 10/mixed lineage kinase 2	MAP3K10/MLK2	22 (KSP)	954	**RP**TT**L**T**F** (JIP-like) [15,68]	2 sites GA**SP**PA**SP**SI (conserved in human)
		mitogen-activated protein kinase kinase kinase 11/mixed lineage kinase 3	MAP3K11/MLK3	27 (KSP KTP)	847		
		mitogen-activated protein kinase kinase kinase 12/dual-leucine-zipper kinase/MAPK-upstream kinase	MAP3K12/MUK/ ZPK/DLK	19	859		
		mitogen-activated protein kinase kinase kinase 13/mixed lineage kinase/leucine zipper-bearing kinase	MAP3K13/MLK/ LZK	16 (KSP)	966		
		mitogen-activated protein kinase kinase kinase 20/MLK-related kinase/human cervical cancer suppressor gene 4	MAP3K20/MRK/ MLTK/ZAKalpha/ HCCZ4	10	800		
		mitogen-activated protein kinase kinase kinase 21	MAP3K21/MLK4	17	1036		
		mitogen-activated protein kinase-binding protein 1	MAPKBP1/MABP1/ JNKBP1	22 (KTP)	1514	**R**AH**L**V**L**D**I** (NFAT4-like) [15,68]	
		serine/threonine-protein kinase TAO1 (MT, actin)	MAP3K16/MARKK /TAO1/TAOK1	8	1001		1 site DPQ**SP**PQV (conserved in human)
		serine/threonine-protein kinase TAO2 (MT, actin, septin)	MAP3K17/PSK1/ TAO2/TAOK2	13	1235		
		serine/threonine-protein kinase TAO3	MAP3K18/DPK/JIK /TAO3/TAOK3	5	898		

Column 3: Human protein names are indicated, with known interlinks between cytoskeletons in red. Column 5: The number of SP and TP sites found in sequences available in UniProt data bank (https://www.uniprot.org) is given for each protein. When at least one KSP/KTP site is present, this is indicated. Column 7: Known JNK substrate sequences are indicated (boxed in yellow, with target sequences in bold and underlined, K amino acids are highlighted in red in KSP and KTP sequences). The positions of the phosphorylated amino acids are

given. When proteins can regulate or be regulated by JNK, this is indicated (green labeling is used to highlight JNK activation). Column 8: Known interactions with JNK are indicated with identified JNK-binding domain (JBD) sequences. Column 9: When SP/TP sites were found phosphorylated in the growth cones of rats in the study of Kawasaki [70], the identified sequences are given including the one conserved in human proteins. K amino acids are highlighted in red in KSP and KTP sequences. Proteins identified in the screen without phosphorylated SP/TP site are marked "no site
